# Oxidation behavior of graphene-coated copper at intrinsic graphene defects of different origins

**DOI:** 10.1038/s41467-017-01814-8

**Published:** 2017-11-16

**Authors:** Jinsung Kwak, Yongsu Jo, Soon-Dong Park, Na Yeon Kim, Se-Yang Kim, Hyung-Joon Shin, Zonghoon Lee, Sung Youb Kim, Soon-Yong Kwon

**Affiliations:** 1 School of Materials Science and Engineering & Low-Dimensional Carbon Materials Center, Ulsan National Institute of Science and Technology (UNIST), Ulsan, 44919 Republic of Korea; 2 School of Mechanical, Aerospace and Nuclear Engineering, Ulsan National Institute of Science and Technology (UNIST), Ulsan, 44919 Republic of Korea

## Abstract

The development of ultrathin barrier films is vital to the advanced semiconductor industry. Graphene appears to hold promise as a protective coating; however, the polycrystalline and defective nature of engineered graphene hinders its practical applications. Here, we investigate the oxidation behavior of graphene-coated Cu foils at intrinsic graphene defects of different origins. Macro-scale information regarding the spatial distribution and oxidation resistance of various graphene defects is readily obtained using optical and electron microscopies after the hot-plate annealing. The controlled oxidation experiments reveal that the degree of structural deficiency is strongly dependent on the origins of the structural defects, the crystallographic orientations of the underlying Cu grains, the growth conditions of graphene, and the kinetics of the graphene growth. The obtained experimental and theoretical results show that oxygen radicals, decomposed from water molecules in ambient air, are effectively inverted at Stone–Wales defects into the graphene/Cu interface with the assistance of facilitators.

## Abstract (web summary)

Graphene holds promise as a protective coating; however, lattice defects may hinder its practical applicability. Here, the authors investigate the oxidation behavior of graphene-coated copper foils and unveil the interplay between structural defects and oxygen radicals from water molecules in ambient air.

## Introduction

Si-based integrated circuits that use Cu instead of Al for interconnections have improved the system performance and power dissipation in ultra-large-scale integration (ULSI) devices, because Cu-based chips have smaller metal components and require less energy to pass electricity because of the low resistivity and high electromigration resistance of Cu compared to those of Al^1,2^. However, the replacement of Al by Cu in interconnections required substantial advancements in fabrication technologies, including Cu patterning methods and the introduction of barrier materials to isolate the Si from potentially damaging Cu atoms. The inability to etch Cu using plasma has led to a new approach of patterning Cu, i.e., the (dual)-Damascene process^3^, which is currently in common use in the semiconductor industry; however, intensive effort is still underway to develop a controllable physical barrier material for Cu in the form of films and coatings ^4–7^. Furthermore, Cu exhibits high reactivity toward oxygen, resulting in the formation of a surface Cu oxide layer, even at room temperature^8^. This layer causes substantial degradation in the interconnection capabilities during processing and/or device operation because of the formation of trap states at the Cu/Cu oxide interface^9–11^. Therefore, the barrier material must completely surround all of the Cu interconnections with a designated thickness, yet possess minimum influence on the physical and electrical properties of the underlying Cu. Many approaches, including coating with metals and alloys such as Ta/TaN and Ru/Ti layers^4,5^ and CuMn and CoW alloy films^6,7^, were concentrically pursued to protect the Cu surface; however, with the scaling down of the feature size to atomic thickness, a stack comprising the aforementioned barrier metal films and a Cu conductor exhibits a greater total resistance than the Al interconnects, eliminating any benefit.

According to the literature, the thickness of the barrier material of Cu should be reduced to < 2 nm to satisfy the requirement of sub−22 nm interconnect technology^1,12^. Thus far, graphene (Gr), the thinnest two-dimensional (2D) material known, appears to be the best candidate to satisfy this requirement because of its unique combination of properties that include ultrathin thickness, high carrier mobility, high chemical and thermal stabilities, and impermeability to all atoms, ions, and molecules^13–19^. Furthermore, Gr/Cu composites have the advantages of conducting a high current density with an enhanced thermal reliability compared to that of bare Cu^20^; in addition, ultrathin graphene films can be directly formed on Cu surfaces with various shapes using a chemical vapor deposition (CVD) process, thereby minimizing any adhesion issues at the interface of graphene and Cu.

However, practically engineered materials are nearly always polycrystalline, and the presence of a certain amount of disorder in crystalline materials is inevitable according to the second law of thermodynamics. In fact, typical films of large-scale graphene produced by CVD are polycrystalline and contain a high density of intrinsic structural defects such as vacancies, adatoms, and line defects^21^. Statistical observations suggest that various topological imperfections in graphene substantially degrade its properties^21–24^; however, the study of the nature of intrinsic graphene defects and their influence on the physical and barrier properties of graphene are still in its infancy. Recently, several pioneering reports have demonstrated that engineered, polycrystalline graphene films can serve as effective diffusion^25–29^ and oxidation^17,30–34^ barriers for Cu. However, the literature contains extensive contradictory results, especially with respect to the effectiveness of graphene coatings as an oxidation barrier for Cu; the detailed mechanism by which Cu is oxidized through a polycrystalline graphene barrier remains a subject of debate^30-36^.

Here, we use optical and electron microscopies to systemically investigate selective oxygen transport through various intrinsic graphene defects in Gr-coated Cu foils. Using an air oxidation process of Gr/Cu composites on a hot plate at ~200 °C, we develop a facile, scalable characterization technique to observe various microscopic topological defects with different origins, such as 0D and 1D defects at nucleation points, intra- and inter-granular grain boundaries (GBs) of graphene grains, multilayer graphene flakes and folded wrinkles in large-area sheets. This technique can be used to correlate the presence and density of intrinsic defects with the properties of macro-scale graphene. The oxidation behavior of Cu through various microscopic graphene defects suggests that the degree of structural deficiencies in as-synthesized graphene can vary depending on the origins of the structural defects, the crystallographic orientations of the underlying Cu grains, the growth conditions of graphene, and kinetics of graphene growth in the CVD reactor. From combined experimental and theoretical studies, we observe that oxygen radicals decomposed from the water molecules in ambient air are the direct cause of the oxidation of Cu and that selective oxygen transport occurs at Stone–Wales (SW) defects through the interface between graphene and Cu with the assistance of facilitators, leaving carboxyl (O = C–OH) groups on graphene as by-products. Finally, we propose a selective oxidation mechanism of Cu through an atomic-scale engineered polycrystalline graphene barrier.

## Results

### Fingerprints of selective oxygen transport in annealed Gr/Cu

**Fig. 1:**
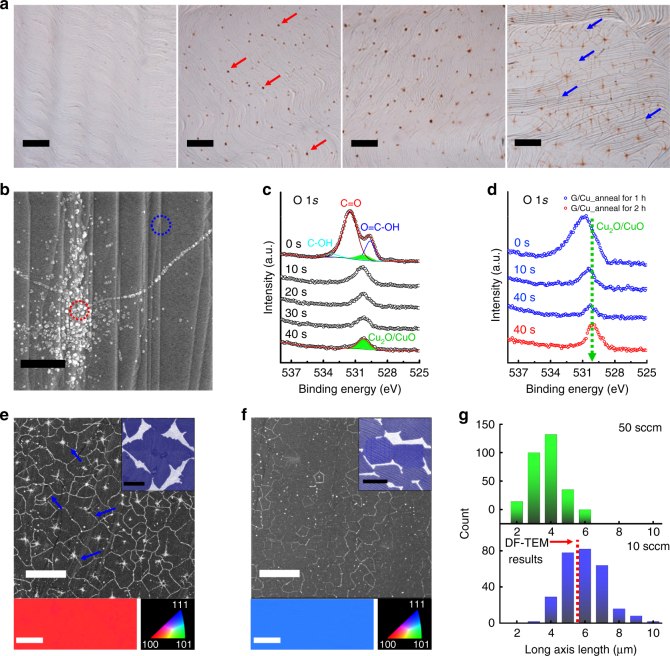
Selective 0D and 1D oxidation fingerprints of fully-grown, air-annealed Gr/Cu samples at low temperatures. **a** OM images of the Gr/Cu sample after annealing at 200 °C for 30, 60, 90, or 120 min (from left to right) in air (scale bar=10 µm). **b** A representative SEM image of the Gr/Cu sample after annealing at ~200 °C for 120 min in air (scale bar=0.5 µm). **c** Depth-profiling HR-XPS O 1*s* line scans of the Gr/Cu sample after annealing at ~200 °C for 120 min in air, depending on the sputtering time. Curve fittings of O 1-*s* spectra were performed using a Gaussian–Lorentzian peak shape after Shirley background correction. **d** Comparison between HR-XPS O 1-*s* peaks of the Gr/Cu samples annealed at ~200 °C for 60 and 120 min in air, depending on the sputtering times. Representative SEM and corresponding EBSD images of a continuous Gr film grown on the grains of **e** Cu(001) and **f** Cu(111); the image was collected after an air oxidation process at ~200 °C for 60 min (scale bar=10 µm). The insets in **e** and **f** show the SEM images of partially grown Gr islands formed on grains of the Cu(001) and Cu(111), respectively (scale bar=2 μm). The bottom images in **e** and **f** show the EBSD images each taken from within one Cu crystal grain with resolution of ~0.1 μm^2^ per pixel (scale bar=3 μm), **g** A change in the long-axis lengths of the polygons formed on the annealed Gr/Cu samples measured by dark-field TEM or SEM, depending on the methane flow rate of 50 (upper) or 10 (bottom) sccm during CVD growth

Graphene films were grown on polycrystalline 25-μm-thick Cu foils at temperatures of ~1000 °C by CVD using a mixture of methane and hydrogen gases; details of the growth procedure are provided in the Methods. The as-synthesized graphene films were of reasonable quality on the basis of structural and optoelectronic measurements (Supplementary Fig. 1). Oxidation was carried out by placing the Gr/Cu samples on a hot plate that was heated to the desired temperature. Figure 1a shows typical optical microscopy (OM) images of the Gr/Cu samples annealed at ~200 °C in air for 30, 60, 90, and 120 min (from left to right). Interestingly, dot features (indicated by red arrows) started to appear and additional line features with a polygon shape (indicated by blue arrows) occurred, when the annealing times were longer than 30 and 90 min, respectively. Notably, the dots were always located at the center of polygons over the whole surface of the samples and the outline of the polygon sharpened as the annealing time was increased. A high-resolution scanning electron microscopy (HR-SEM) image of the Gr/Cu sample after 120 min of annealing (Fig. 1b) shows the presence of Cu surface steps, graphene wrinkles, and the presence of dot and line features. In particular, the dot and line features consisted of globular-shaped nanoparticles and were observed to be bulged out from the surface; however, other regions retained their original Cu surface morphologies. We therefore further investigated the presence and chemical states of foreign species in the annealed Gr/Cu samples using energy dispersive X-ray spectroscopy (EDS). A typical SEM-EDS spectrum of the samples shows two substantial Cu peaks throughout the samples at binding energies of 0.95 and 0.83 keV, which correspond to Cu L_α_ and Cu L_β_, respectively (Supplementary Fig. 2). However, a weak O K_α_ signal was detected at a binding energy of ~0.53 keV only in the areas of dot and line features (denoted by red circle in Fig. 1b), indicating that selective oxidation of the Gr/Cu surface occurred along the dot and line features.

To check if the underlying Cu surface was oxidized, the compositional changes in the samples with depth were investigated by depth-profiling X-ray photoelectron spectroscopy (XPS) paired with Ar^+^ ion sputtering. The graphite-like *sp* ^2^-hybridized C peak at ~284.4 eV in the HR C 1*s* line-scan spectra^37^ gradually diminished with a chemical shift to lower binding energy as the sputter time increased and then finally disappeared after 40 sec sputtering (Supplementary Fig. 3a), implying that the graphene layer was completely etched away^38^. From the HR O 1s line-scan spectra, it was found that the O atoms exist in four different forms based on their binding energy: hydroxyl (C–OH) at 533.3 eV (4.7 at% of O atoms), carbonyl (C = O) at 531.4 eV (67.6 at% of O atoms), Cu_2_O/CuO phase at 530.3 eV (8.1 at% of O atoms), and carboxyl (O = C–OH) at 529.5 eV (19.6 at% of O atoms) (Fig. 1c); however, the oxygenated groups were completely removed after 10 s of sputtering and a dominant peak at ~530 eV corresponding to the Cu_2_O/CuO phase^39^ (denoted by the green area in Fig. 1c) was constantly observed for up to 40 s of sputtering, in contrast to the Gr/Cu samples without dots and line features (Supplementary Fig. 3b–d). This finding indicates that the dot and line features are a representative mark of a selective oxidation that occurred in the underlying Cu after the annealing process. Furthermore, the levels of the Cu_2_O/CuO peak substantially increased as a result of prolonged annealing from 60 to 120 min at 200 °C, as shown in Fig. 1d. These results confirm that the dot and line features were indeed the result of the air oxidation of the Gr/Cu samples.

We performed a closer examination of the oxidation line features in the annealed Gr/Cu samples as a function of the crystallographic orientation of the underlying Cu grains. Notably, commercial Cu foils were polycrystalline materials and the Cu(100) grain was the most favorable component of both the as-received and annealed Cu foils with volume fractions of ~91% and ~74%, respectively. Figure 1e, f displays a representative SEM image and a corresponding electron-backscatter diffraction (EBSD) mapping of the continuous graphene films grown on grains of Cu(100) and Cu(111), respectively, following the air oxidation process at 200 °C for 60 min. The primary shapes of the oxidation line features were similar to those of the graphene islands partially grown on each Cu grain (insets of Fig. 1e, f); the oxidation line feature on the Cu(100) grains was predominantly a four-lobed shape with a long-axis length of ~5.5 ± 1.2 μm (Fig. 1e), whereas that on the Cu(111) grains was a hexagonal shape with a long-axis length of ~2.8 ± 0.7 μm (Fig. 1f). We note that the long-axis lengths of the oxidation line features in both grains were analogous to the average sizes of the graphene islands grown on each Cu grain (based on dark-field TEM observations (bottom in Fig. 1g and Supplementary Fig. 4)), suggesting that the oxidation process is dominantly favored at the inter-granular GBs of graphene. We investigated the changes in the long-axis length of the oxidation line features by controlling the size of the graphene islands. The size of the graphene islands was decreased with increasing methane flow rate because of the high nucleation density^40,41^; thereby, the long-axis length of the line features decreased to ~3.1 ± 0.6 μm at a methane flow rate of 50 sccm (top in Fig. 1g). Interestingly, we observed that the oxidation line features that formed on the Cu(100) grains were thick and clearly visible compared to those on the Cu(111) grains. This phenomenon reflects the strong influence of the Cu crystal orientation on the oxidation resistance of the graphene films and demonstrates that the introduction of the Cu(111) grains leads to increased oxidation resistance of the resulting graphene films because of a better level of atomic stitching between the connecting GBs^42,43^. We occasionally observed the presence of intra-granular GBs within the polygon-shaped line features (blue arrows in Fig. 1e) on the Cu(100) grains; these GBs originated from the multi-domain nature of the graphene islands^44^. The intra-granular GBs on the Cu(100) grains can be fully revealed throughout the whole surface by increasing the annealing time up to 240 min (blue circles in Supplementary Fig. 5). This accounts for intra-granular GBs being more resistant to oxidation than inter-granular GBs.

### Oxidation behavior of Cu through various graphene defects

**Fig. 2:**
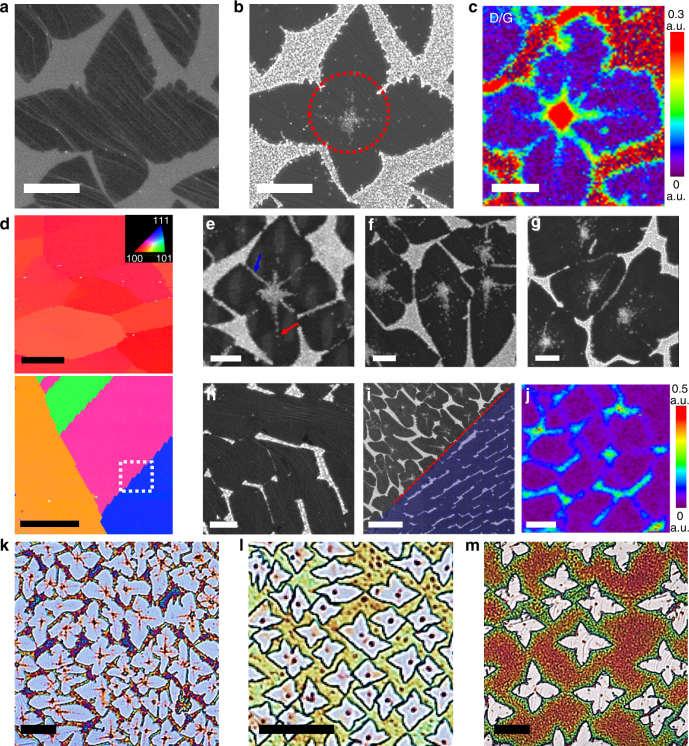
Different oxidation behaviors of various microscopic graphene defects in graphene islands depending on variables. Representative SEM images of Gr islands grown on a Cu foil at 1000 °C for 1 min **a** before and **b** after annealing at ~200 °C for 70 min in air (scale bar=3 µm). **c** A typical Raman D/G map image of the as-synthesized Gr islands transferred onto SiO_2_/Si (scale bar=3 µm). **d** Typical EBSD map images of the Cu foil after Gr islands were grown at 1000 °C, followed by the annealing process at ~200 °C for 120 min in air (scale bar=50 µm). The EBSD data show that the Cu foils consist of grains mostly with orientations of approximately (100) (upper) and, in some regions, grains with orientations of (111) and (101), as well as high-index orientations (bottom). **e**–**i** Representative SEM images of the annealed Gr islands grown on Cu grains with an orientation of **e**, **f** (100), **g** (101), **h** (111), and **i** the boundary region between (100) and (111) (indicated by the white-dotted square in Fig. 2d). Scale bars, 2 µm **e**–**h**, 10 µm **i**. **j** A typical Raman D/G map image of the Gr islands grown on a Cu(111) grain transferred onto SiO_2_/Si (scale bar=2 µm). **k**–**m** OM images of the Gr islands grown on Cu(100) grains after annealing at ~200 °C for 120 min under various CVD growth conditions. The Gr islands were grown **k** at ~1050 °C for 30 s under CH_4_ (10 sccm) and H_2_ (5 sccm), **l** at ~1000 °C for 30 s under CH_4_ (10 sccm) and H_2_ (20 sccm), and **m** at ~1,000 °C for 3 min under CH_4_ (2 sccm) and H_2_ (5 sccm). The scale bars represent 10 μm

We halted growth before the graphene islands merged with each other to form a continuous graphene film. Figure 2a, b shows representative SEM images of an isolated graphene island on a Cu(100) grain before and after air oxidation at ~200 °C. As a result of the oxidation, the white dot assemblies appeared both in the center (red circle in Fig. 2b) and in the exterior, i.e., at the surface of the graphene islands and at the surface of a bare Cu grain, respectively. From HR-SEM images acquired from the samples (Supplementary Fig. 6), we note that there was no significant difference in the morphologies of the globular-shaped nanoparticles formed in both regions and the size of oxide nanoparticles is in a similar range of ~30 to ~50 nm. From many literatures^45–47^, it is well known that the Cu oxides formed after oxidation at low temperatures below 300 °C generally have fine crystals composed of three regions: a thin CuO layer, a thick Cu_2_O layer, and an oxygen-containing Cu region, and the volume change associated with the solid-state transformation at the CuO/Cu_2_O interface produces compressive stresses during the oxide growth, which stimulate the formation of fine oxide particles to accompany the interface reaction.

A closer examination of the center revealed that the white dots possessed two overlapping features: rhombus-shaped and cross-shaped dot assemblies. To explore the nature of the dot assemblies, the as-synthesized graphene islands without any oxidation process were transferred onto SiO_2_/Si(100) substrates for Raman spectroscopy. The intensities of the characteristic graphene Raman peaks, D (~1351 cm^−1^), G (~1588 cm^−1^) and 2D (~2685 cm^−1^), were extracted^48–50^; their spatial dependences are plotted in Fig. 2c and Supplementary Figs. 7 and 8. The typical Raman D/G band map in Fig. 2c indicates the presence of two types of structural defects in the center of the graphene islands. The typical *I*_D_/*I*_G_ intensity ratio was negligibly small (<0.05) over most of the area within the graphene islands, with the notable exception of the central area displaying a relatively large *I*_D_/*I*_G_ related to the rhombus-shaped (*I*_D_/*I*_G_ ≈ 0.3) and cross-shaped (*I*_D_/*I*_G_ ≈ 0.2) dot assemblies. We mapped numerous (>30) graphene islands and observed that every island contains two such overlapping features in the center, regardless of the size of the graphene islands grown under various growth conditions (Supplementary Fig. 8). We note that a pronounced *I*_D_/*I*_G_ ratio was also observed on the edges of the islands; however, the magnitude of *I*_D_/*I*_G_ in the rhombus-shape dot assemblies was always higher than that in the other defective regions including the intra-granular GBs and the edges of the islands, implying that the air oxidation process provides a convenient means to clearly identify the locations of intrinsic graphene defects and to discern the degree of structural deficiencies. Furthermore, our observation of a large *I*_D_/*I*_G_ at the white dot assemblies in the SEM images suggests that an improvement of the crystalline quality of the early graphitic nuclei during/after nucleation events is necessary to obtain high-quality, large-area graphene sheets.

Figure 2d–i presents typical SEM-EBSD mapping images taken from representative graphene islands with respect to the crystallographic orientations of the underlying Cu grains. After the graphene growth, the Cu(100) grain (with a volume fraction of ~74%) was still the most favorable component of the Cu foil; in some regions, the Cu foil had different crystallographic orientations such as Cu(111), Cu(101), and high-index orientations, as shown in Fig. 2d. For Cu grains with orientations of ~(100) and (101), the white dot assemblies at the nucleation points were always observed in nearly all graphene islands (Fig. 2e–g), whereas no oxidation line features were observed on the Cu grains with orientations of ~(111) (Fig. 2h). This dependence on the crystallographic lattice orientation of Cu was clearly exposed at the boundary region between Cu(100) and Cu(111) grains (Fig. 2i). We note that, in graphene islands spanning across Cu(100) and Cu(111) grains, the oxidation line features running from the nucleation center formed on the Cu(100) were precisely blocked at the intersection of the Cu(100) and Cu(111) grains (red dotted line in Fig. 2i). From these observations, we observed that the structural properties of the graphene nucleation regions on the Cu(100), Cu(101) and high-index surfaces were substantially inferior to those on the Cu(111) surface. This observation was further confirmed through a Raman map of the D/G ratios of the transferred graphene islands grown on Cu(111) grains. As shown in Fig. 2j, the D/G ratio at the nucleation point on the Cu(111) grains was dramatically reduced. These results clearly indicate that the structural quality of the graphene islands at the nucleation point was strongly dependent on the crystallographic orientation of the underlying Cu grain. Given this effect, the growth of graphene on the Cu grains with orientations of ~(111) is a promising approach to achieve graphene with high structural quality.

In the next observation, the cross-shaped oxidation line features centered at the nucleation point were distinctively characteristic of the graphene grown on the Cu(100) grains, which were different from the intra-granular GBs presented between lobes (blue arrow in Fig. 2e). Because the cross-shaped lines correctly aligned to the apex of each symmetric lobe (red arrow in Fig. 2e) and had different lengths in asymmetric lobes (Fig. 2f), we inferred that the cross-shaped line features directly correlate with the graphene growth kinetics on the Cu(100) surface. Thus, we more closely examined the cross-shaped line features and their dependence on the growth rate of graphene islands by controlling the growth conditions, such as growth temperature and gas flow rates during the CVD process. In the case of graphene islands grown with a higher growth rate by increasing the growth temperature from ~1000 to ~1050 °C, the cross-shaped line features formed at the nucleation points of nearly all of the graphene islands (Fig. 2k). However, by introducing a lower growth rate through only increasing the hydrogen flow rate from 5 to 20 sccm (Fig. 2l) or only decreasing the methane flow rate from 10 to 2 sccm (Fig. 2m), we observed that the cross-shaped line features nearly disappeared and that the Gr/Cu samples were oxidized only at the central nucleation points. These observations demonstrate that, with a high growth rate, the early graphitic nuclei contained many structural defects such as vacancies, voids, or pores; thus, the introduction of a low growth rate, at least during the nucleation events, will enhance the crystalline quality of the resulting graphene films.

**Fig. 3:**
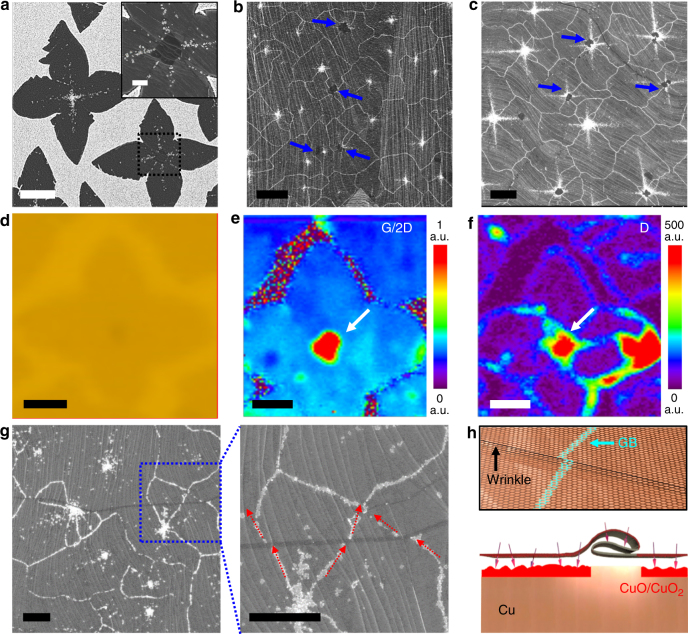
Structural characterization of the oxidation behavior of Cu at the presence of multilayer graphene flakes and folded wrinkles. **a** A representative SEM image of Gr islands grown on a Cu(100) grain, followed by annealing at ~200 °C for 120 min in air. The inset is a higher magnification SEM image of the black-dotted square in a (scale bar=1 μm). Typical SEM images of the fully grown Gr/Cu sample after annealing at ~200 °C in air for **b** 120 and **c** 240 min. Blue arrows in b and c indicate the nucleation points of Gr covered by multilayer flakes. **d**–**f** An OM image and corresponding Raman G/2D and D map images of a Gr island with a multilayer flake in the center, after being transferred onto SiO_2_/Si (from left to right). White arrows in e and f indicate the position of a multilayer Gr flake. **g** A typical SEM image of the fully grown, air-annealed Gr/Cu sample containing folded wrinkles, after annealing at ~200 °C in air. The right is a higher magnified SEM image of the blue-dotted square, and the red arrows indicate the presence of a kink in the oxidation line features at the folded-wrinkle regions. Scale bars=5 µm **a**–**c**, 2 µm **d**–**g**. **h** Schematic diagram represents the effective anti-oxidation mechanism of Cu because of the presence of folded wrinkles in Gr

Another key finding is the disappearance of the rhombus-shaped white dot assemblies at the center of some graphene islands grown on Cu(100) grains, as shown in Fig. 3a. We mapped several (> 100) graphene islands by HR-SEM and observed that a multilayer graphene flake always existed at the center when the rhombus-shaped white dot assemblies were absent; the multiple layers appear as the darker region in the SEM image because of the reflection of fewer secondary electrons^51^ (inset of Fig. 3a). This phenomenon of multilayer flakes protecting the underlying Cu against oxidation was also observed in the fully grown Gr/Cu samples annealed for 120 min (blue arrows in Fig. 3b); even when the annealing time was increased to 240 min, this ability to inhibit the oxidation of the underlying Cu surface was retained (blue arrows in Fig. 3c). Notably, the central nucleation points of the graphene grains were still defective despite the presence of the multilayer flakes, as confirmed by the Raman map images of *I*_D_/*I*_G_ and D band in Fig. 3d–f; however, the oxidation process of the Cu surface was inhibited because of the presence of the multilayer graphene flakes. Given the very high diffusivity (~3.8 × 10^−5^ cm^2^ s^−1^) of molecular oxygen (O_2_) between graphitic layers^52^, these observations strongly suggest that the oxygen molecules present in air were not a major cause of the oxidation of the Cu surface. Similarly, careful observations revealed that the white line features in the fully grown, annealed Gr/Cu samples always exhibited a kink when they met the folded wrinkles in graphene, as shown in Fig. 3g. This observation indicates that the multilayer structure was induced by the morphological transition of the tall ripple into a folding system to minimize the total energy and was not oxidized after annealing in air. As illustrated in Fig. 3h, the Cu surface under the folded wrinkles was effectively protected from oxidation; thus, the oxidation lines along the GBs ceased at the folded wrinkles.

### Oxidation mechanism of Cu through graphene defects

**Fig. 4:**
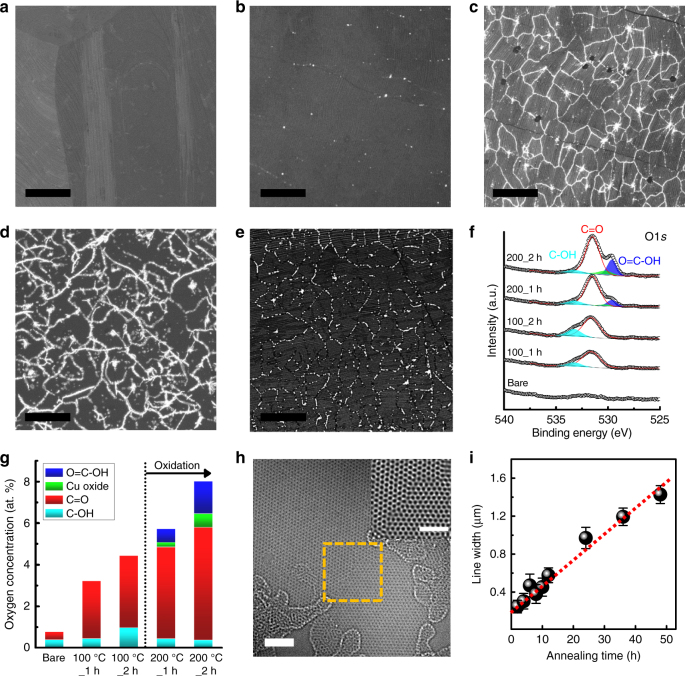
Control experiments to elucidate the origin and process of Cu oxidation through a polycrystalline graphene barrier. Typical SEM images of fully-grown Gr/Cu samples annealed at ~200 °C **a** for 120 min under vacuum (P ≈ 10^−3^ Torr), **b** for 120 min in a pure O_2_ atmosphere (P ≈ 150 Torr), **c** for 120 min in ambient air (RH ≈ 30–40%), **d** for 60 min in a wet-O_2_ atmosphere (RH ≈ 80%), and **e** for 60 min in a wet-N_2_ atmosphere (RH ≈ 50%). The scale bars represent 10 µm. **f**, **g** A comparison of the HR-XPS O 1s peaks and of the chemical compositions of the Gr/Cu samples depending on the annealing temperature and time in ambient air. **h** Representative plan-view HR-TEM image of a Gr film transferred onto a TEM support grid (scale bar: 2 nm). The inset is an atomic-resolution image of a GB of Gr, indicated by a yellow square (scale bar=0.5 nm). **i** Plot of the average width of the oxidation line feature as a function of annealing time up to 48 h based on the SEM observation. Error bars represent ±1 standard deviation, obtained from 100 different points in each sample

To elucidate the origin of the selective oxidation process of Cu through graphene defects, we examined the variation of the oxidation behavior in five different annealing environments at ~200°C, as shown in Fig. 4a–e. Interestingly, the fully grown Gr/Cu samples annealed under vacuum (*P* ≈ 10^−3^ Torr, Fig. 4a) and in a pure O_2_ atmosphere (*P* ≈ 150 Torr, Fig. 4b) were not oxidized through various graphene defects such as the intra- and inter-granular GBs and nucleation points of the graphene grains, in contrast to the same Gr/Cu sample annealed in air (Fig. 4c). From the control experiments, we demonstrated that molecular oxygen (O_2_) itself was not a direct cause for the oxidation phenomenon of Cu and that earlier observations in our study were irrelevant to the segregation of oxygen species that intrinsically resided in the bulk of Cu during the annealing process^37^. Previous theoretical and experimental studies on the dissociative adsorption of O_2_ and H_2_O on graphene with or without various defects^53,54^, have suggested that O_2_ has relatively high dissociative reaction barriers of ~1.78 and ~2.80 eV on a pristine graphitic lattice and at the GBs of graphene grains, respectively, except for the di-vacancy region (~0.42 eV); by contrast, H_2_O molecules on a defective graphitic lattice such as edge (~0.06 eV), mono-vacancy (~0.04 eV) or di-vacancy (~0.35 eV) regions can be easily spilt into hydrogen and hydroxyl radicals, even at room temperature. We therefore performed oxidation experiments in a wet O_2_ atmosphere (relative humidity (RH) ≈ 80%, Fig. 4d) to further investigate the role of H_2_O and observed that the oxidation line features using wet O_2_ were significantly thick and clearly visible compared to those using ambient air (RH ≈ 30-40%, Fig. 4c) under the same annealing condition of the Gr/Cu samples. In addition, we have performed an annealing process of the Gr/Cu samples in a wet N_2_ atmosphere (RH ≈ 50%, Fig. 4e) to minimize any oxygen exposure of the samples. Similar to the Gr/Cu samples annealed in air and in a wet O_2_ atmosphere, the oxidation line features through various graphene defects were clearly visible from OM and SEM images (Supplementary Fig. 9a, b) in the Gr/Cu samples annealed in a wet N_2_ atmosphere. Interestingly, we found that the degree of oxidation was significantly altered in different gas ambients, as observed in many thin-film formation and phase transformation processes^55,56^. As expected, no oxidation features were found in the Gr/Cu samples annealed in a pure N_2_ atmosphere (Supplementary Fig. 9c). This result reflects the strong influence of the H_2_O on the oxidation phenomenon and demonstrates that the water molecules in air play a key role in the selective oxidation of Cu through intrinsic graphene defects of different origins.

Figure 4f shows typical XPS results for a bare Cu foil and fully grown Gr/Cu samples annealed at ~100 and ~200 °C for 60 and 120 min in air. OM and SEM observations revealed that the various oxidation features appeared only in the samples annealed at ~200 °C; the samples annealed at ~100 °C did not exhibit any indication of oxidation. Interestingly, the XPS spectra of all of the annealed Gr/Cu samples showed substantial O 1s peaks at 530 and 533 eV, corresponding to carbonyl (C = O) and hydroxyl (C–OH) groups, respectively. Unlike the unoxidized samples, significant amounts of carboxyl (O = C–OH) groups were detected in the case of the samples annealed at ~200 °C (Fig. 4g), indicating that the carboxyl (O = C–OH) group present on graphene was a major by-product of the oxidation process that occurred in the moisture-rich environment. Thus, we infer that the oxygen radicals dissociated from H_2_O molecules on graphene will selectively penetrate through the graphene defects such as vacancies or pores. We mapped multiple (> 20) graphene grains with an aberration-corrected HR-TEM; however, detecting the presence of vacancies or pores in our graphitic frameworks was very difficult. Instead, we dominantly observed the graphene GBs consisting of alternating pentagon and heptagon carbon rings, i.e., a chain of SW-like bond rotations (Fig. 4h).

So, a key question is how the underlying Cu is oxidized through the graphene GBs, which do not involve any removed atoms. To answer this question, we performed spin-polarized density functional theory (DFT) calculations^57,58^ on the selective transport of O atoms through a bare graphene without Cu substrate and on their dependence on the attached functional groups to understand the origin of the oxygen inversion process (Details of the calculations are presented in the Methods). We obtained an inversion energy barrier of ~2.46 eV for an epoxide (C-O-C) at the SW defect, and this energy barrier was dramatically diminished when H, OH, or O atoms were attached to sites around the epoxide, as shown in Table 1 and Supplementary Fig. 10. We note that the inversion energy barrier of ~0.31 eV was achieved when an additional O atom was attached to the epoxide, implying that the selective oxygen-transport through the SW defect can occur even near room-temperature. The reason that the diffusion of an O atom that is assisted by an additional O atom has the lowest barrier can be clearly shown by the Supplementary Fig. 11. For more accuracy, we have further calculated the lowest barrier energy including a slab of Cu to investigate the effect of Cu substrate (details of the calculations are presented in the Methods). As shown in Supplementary Fig. 12, the slab of Cu seems to hinder the diffusion of an O atom through the SW defect of graphene resulting in a higher energy barrier (~0.7 eV) than the case of a bare graphene without Cu substrate (~0.31 eV). However, we note that this result won’t affect our conclusion that the selective oxygen transport through the SW defect can occur even near room-temperature. More importantly, the free energy difference between the reactants and products (Δ*G*) after oxygen inversion process through the SW defect is around 0.1 eV, indicating that the inversed epoxide is thermodynamically stable in the Gr/Cu system. Furthermore, considering the fact that a bond breaking energy of the carbon dioxide on Cu is ~1.0 eV^59^, the inversed epoxide is expected to be more easily dissociated on Cu in our experimental conditions because the epoxide has a single bond (C-O), resulting in the selective oxidation of the underlying Cu through graphene GBs.

**Table 1:** Calculated inversion barrier of an oxygen atom at a SW defect through free-standing graphene as a function of the types of attached functional groups

Functional group	Oxygen inversion barrier (eV)
without	2.461
1 H	1.700
2 H	1.573
1 OH	1.484
2 OH	1.502
1 O	0.314

Here, it is worthwhile discussing on where oxygen radicals, which penetrate graphene through the SW defects on GBs and finally oxidize the Cu surface, are generated. One possibility is the direct dissociation of H_2_O molecules at GBs, but it may be hardly possible because the energy barrier for the dissociation (~2.71 eV) is too high^54^. Instead, we have studied the possibility that the dissociation takes place at other defected sites on graphene. Previous experimental and theoretical studies have indicated that intrinsic defects are always present and stabilized in typical films of large-area graphene produced by CVD and that they can catalyze the dissociation of H_2_O molecules in ambient air; this dissociation can even occur at room temperature given the very low dissociation energy barriers of the H_2_O molecule on various intrinsic defects such as edge (~0.06 eV), mono-vacancy (MV) (~0.04 eV) and di-vacancy (~0.35 eV) regions^53,54^. For instance, we have performed spin-polarized DFT calculations on the configuration of the H_2_O molecule on a graphene MV, as shown in Supplementary Figs. 13 and 14 (details of the calculations are presented in the Methods). Our theoretical calculations indicate that a H_2_O molecule is dissociated to one O and two H atoms on a graphene MV and that both the dissociated O and H atoms can diffuse laterally with the energy barriers of ~1.9 eV. Although the energy barriers for O and H atoms for lateral diffusion from the MV are still high, they are significantly lower than the dissociation energy barrier of H_2_O at GBs (~2.71 eV)^54^. Once the O and H atoms dissociated at the graphene MV diffuse laterally to non-defected sites (to outward positions), the graphene MV can return to its original configuration, implying that the graphene defects/vacancies will produce O radicals repetitively in a catalytic manner. Importantly, we found that the presence of the underlying Cu greatly reduces the activation barriers of both the dissociated O and H atoms into neighboring C rings. We do not rule out the possibility of the dissociation of H_2_O at graphene GBs, but it is reasonable to expect that O radicals, which penetrate the graphene through the SW defects, come from the defected sites such as vacancies or edges after the dissociation of H_2_O molecules. In addition, the resulting atomic oxygen, which escapes from the defected sites, diffuses on perfect graphene with relatively low barriers (mostly < 1 eV), although its actual diffusion rate is different depending on the local environment^60,61^. Furthermore, the atomic oxygen can readily diffuse on graphene with Cu substrate compared with a pristine graphene surface without Cu substrate because of the spontaneous electron doping of graphene by Cu^62,63^. Finally, upon reaching the GBs, the O atoms accumulate because the high barrier (~1.4 eV)^61^ for reverse displacement should ensure their stability at low temperatures. From all this we may conclude that the oxygen radicals are generated more frequently by the dissociation of H_2_O molecules at defected sites of graphene rather than at graphene GBs. And, they diffuse and accumulate on graphene GBs and then penetrate the graphene through SW defects with the assistance of facilitators.

**Fig. 5:**
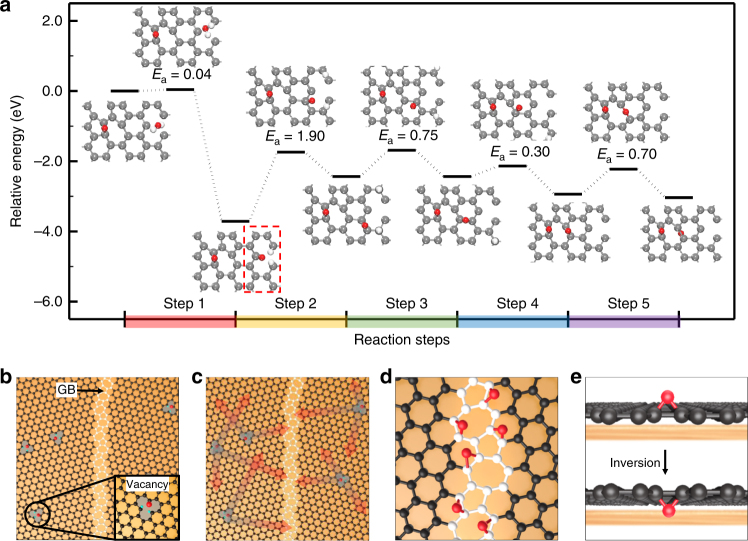
Reaction pathway and schematics of the entire oxidation processes. **a** Minimum energy pathway of the entire reaction of oxidation (energy barriers (*E*_a_) in eV); started from the dissociation of water molecules at MVs and completed by the inversion of radical O atoms at GBs of graphene on Cu(111) substrate. The gray, white and red spheres indicate the C, H and O atoms, respectively. The diagrams represent the elementary steps of the Cu oxidation process including **b** the dissociative chemisorption of water vapors at point defects in Gr, **c** the migration of O adatoms on the Gr/Cu surface, **d** the accumulation of O atoms at the GBs of the Gr grains, and **e** the inversion of the O atoms at a SW defect with additional functional groups, followed by the selective oxidation of the Cu surface

In Fig. 5, we summarized the entire oxidation mechanism we have discussed so far. The entire oxidation process consists of a series of successive reactions, as displayed in Fig. 5a. The stated barrier energy in Fig. 5a is based on the reaction of O atom. The optimized configurations and corresponding reaction barriers are shown for each reaction step. In Step 1, the dissociation of water molecules in air takes place at the graphene MVs with the energy barrier of ~0.04 eV, resulting in a carbonyl group (O = C) (marked by the red dashed line in Fig. 5a) and two C–H configurations^54^. In Step 2, the dissociated H and O atoms diffuse laterally from the MV defect site to the six-ring configuration of graphene with the energy barrier of ~1.9 eV each. The H atom is bonded at the top of the C atom and the O atom is stabilized in the middle of the C atoms. In Step 3, the H and O atoms continue their lateral diffusion from one stable six-ring to a neighboring six-ring configuration with the energy barriers of ~0.3 eV^64^ and ~0.75 eV^60^, respectively. In Step 4, the accumulation of O atoms at SW defects of graphene is relatively preferred by the diffusion of O atom from perfect hexagon to graphene GBs with the forward and backward energy barriers of ~0.3 and ~0.8 eV, repectively^61^. Finally, in Step 5, the inversion of the O atom through the SW defects on GBs takes place with the assistance of additional O atoms, with the energy barrier of ~0.70 eV. We note that the suggested oxidation mechanism clearly shows a catalytic cycle of H_2_O splitting at the graphene defect sites.

In addition, we examined the possibility that other functional groups join the oxidation mechanism, especially after the dissociation process of a water molecule at graphene MVs (see Supplementary Fig. 15 and Supplementary Note 1). For this, we considered the carboxyl (O = C–OH) and hydroxyl (C–OH) groups, which were abundantly detected by XPS in the experiment (Figs. 1c and 4f). Our calculations showed that the carboxyl and hydroxyl groups have stable configurations at MV sites like carbonyl groups, and thus there is some possibility that carboxyl and/or hydroxyl groups may either hinder or decelerate the further oxidation reactions. By conducting nudged elastic band (NEB) calculations, we found that the lateral diffusion of both functional groups was impossible, but they could be further transformed to other configurations with some energy barriers. The carboxyl group could be decomposed to a carbonyl group and an OH molecule with a ~0.72 eV energy barrier. The dissolved OH molecule can diffuse outward from the MV site with a ~0.5 eV energy barrier^65^, and therefore we can expect that the final state of the carboxyl group returns to the same configuration in which a carbonyl group and two H atoms exist, which results from the direct dissociation of a water molecule at MVs. This means that forming a carboxyl group at a graphene MV site is energetically stable, but it can transform to a carbonyl group and thus can further contribute to oxidation processes. Thus, we may conclude that the formation of a carboxyl group does not halt the oxidation process. In other words, the oxidation process is either hardly affected by the formation of carboxyl group or is affected within an allowable margin. Similarly, we found that the stable hydroxyl group at graphene MV can only contribute to the oxidation process after it has transformed to a carbonyl group and a single H atom. It is noticeable that the possibility that the formation of hydroxyl groups decelerates the oxidation process was higher than that in the case of carboxyl groups because of a relatively high diffusion barrier (~1.23 eV) of the H atom (see Supplementary Fig. 15).

Our work suggests that the new oxide formation occurs at the Gr/Cu interface as a result of the penetration and dissociation of O atoms. Using aberration-corrected HR-TEM, we find the change in the atomic configuration of the graphene GBs and the presence of under-coordinated edge C atoms, e.g., vacancies and/or the alternating 5- and 9-membered chain, after the oxidation process; however, we note that the GBs are not broken in all investigated regions under the TEM (Supplementary Fig. 16). We suggest that the change in the atomic configuration of the graphene GBs may be originated from the high in-plane tensile load applied in graphene because of the large difference in the molar volumes of the Cu and oxides during the phase transformation^45–47,66^. The major oxidation products such as carbonyl (C = O) and carboxyl (O = C–OH) groups on graphene are expected to be present at defective regions in graphene, e.g., the under-coordinated C atoms in graphene GBs since they possess dangling bonds^67,68^. It is interesting to note that the O/C ratio of the Gr/Cu samples after air oxidation at ~200 °C for 120 min corresponded approximately to the estimated fraction of intrinsic defects in graphene (see Supplementary Fig. 17 and Supplementary Note 2).

To further examine the role of the carboxyl (O = C–OH) group present on graphene in the oxygen transport, we carried out the controlled oxidation experiments of the Gr/Cu samples at ~200 °C as a function of annealing time. The average width of the oxidation line features shows a strong dependence on the annealing time, increasing linearly from ~0.25 to ~1.40 μm with increasing the annealing time from 2 to 48 h, as shown in Fig. 4i, indicating that the underlying Cu surface is consistently oxidized up to 48 h (The corresponding OM and SEM images of the oxidation line features at different annealing time are shown in Supplementary Fig. 18).

We further conducted NEB and DFT calculations to examine the stability of carboxyl groups at defects other than MVs and the possibility that they penetrate at SW defects. For the stability of carboxyl groups, we considered the disconnection between C atoms, so-called unzipping, instead of vacancies for two cases: perfect six-ring and SW defect structures of graphene, as shown in Supplementary Fig. 19 and Supplementary Note 3. In both cases, two of C-C bonds in graphene are broken first and energetically stable carboxyl groups can be formed even at the perfect six-ring and SW defects of graphene. These carboxyl groups can be transformed to the more stable O–C–OH configuration with the calculated transformation energies of ~1.18 eV and ~0.54 eV for the unzipped six-ring and SW defects, respectively. In addition, the estimated inversion barrier of an O atom from the O–C–OH configuration through an unzipped SW defect of graphene was too high at ~3.9 eV. The calculation results so far support two facts. One is that the carboxyl group can be formed because they are energetically stable at MVs (Supplementary Fig. 15b) as well as at unzipped six-ring or SW defects (Supplementary Fig. 19a) of graphene; It can be a proper answer to why abundant carboxyl groups were detected by XPS in the experiment. The other is that the oxidation from carboxyl groups hardly takes place because they have to be transformed to the carbonyl structure again at MVs (Supplementary Fig. 15b) or the inversion barrier of the O atom is too high at SW defects (Supplementary Fig. 19b) of graphene. Therefore, our experimental and computational results strongly suggest that the presence of carboxyl groups on graphene does not significantly disturb the oxygen transport.

## Discussion

The suggested selective oxidation mechanism of Cu with a polycrystalline graphene barrier is schematically illustrated in Fig. 5b–e. Eventually, selective oxidation fingerprints of Cu formed at various microscopic graphene defects such as 0D and 1D defects at nucleation points, the intra- and inter-granular GBs of graphene grains, multilayer graphene flakes and folded wrinkles in large-area sheets. Using a combination of simple microscopy techniques and DFT calculations, we observed that water molecules in ambient air are a direct cause of the selective oxidation phenomenon of Cu and that the oxidation resistance of structural deficiencies in graphene varies depending on the origins of the structural defects, the crystallographic orientations of the underlying Cu grains, and the growth conditions of graphene, and the kinetics of the graphene growth in the CVD reactor. Our results are readily applicable to correlating the presence and density of various microscopic graphene defects with the properties of macro-scale graphene films, thereby advancing the commercialization of such films. This ability to easily access the location and discern the oxidation resistance of various graphene defects in large-area sheets addresses a substantial challenge in the development of advanced graphene barriers for interconnections, advanced metallization, and other practical applications across many industries. We believe that our methodology is not only limited to the graphene films used in this study, and we expect that similar process strategies can be developed for emerging other 2D nanomaterials and their heterostructures. Further investigations of microscopic defect healing in polycrystalline graphene of large-area sheets using this methodology are necessary to enhance the performance and reliability of Gr-based device.

## Methods

### Synthesis of graphene on a Cu foil

A homemade hot-walled CVD system was used to grow graphene on 25**-**μm-thick Cu foils (Alfa Aesar, 99.8% purity) that had been previously cut with razorblade into 4 × 4 cm^2^ strips. These strips were electrochemically polished in phosphoric acid for 15 min to clean the bare Cu surface and were subsequently rinsed with distilled water followed by isopropyl alcohol (IPA). Immediately after the cleaning process, the Cu strips were loaded into a 4 in. quartz tube followed by evacuation of the chamber to ~3 mTorr; the temperature was then increased to 1000–1050 °C under an H_2_ atmosphere (H_2_ flow rate of 5 sccm). After the Cu strips were annealed for 10 min, the graphene was synthesized by introducing methane gas (CH_4_ flow rate of 10 sccm) and then cooling the Cu strips under the same conditions. Following the growth, the graphene layers were transferred onto SiO_2_/Si for further investigation using a conventional wet-transfer method assisted by a poly(methyl methacrylate) (PMMA) supporting layer and an aqueous solution of 1M ammonium persulfate.

### Raman spectroscopy and mapping

The Raman spectroscopy and mapping were carried out on a WiTec Alpha 300R M-Raman system equipped with a computer-controlled *x*–*y* translation stage and a 532 nm excitation source; the laser spot had a diameter of ~640 nm for a ×50 objective lens with NA= 0.5. During the measurements, the laser power was ~1 mW at the sample to avoid laser-induced thermal effects or damage. The Raman spectra and mapping images were analyzed using the WiTec Project software.

### X-ray photoelectron spectroscopy (XPS)

The XPS studies were conducted on a K-alpha spectrometer (Thermo Fisher) using a non-monochromatic aluminum Kα X-ray excitation source operated at a power of 72 W; the diameter of the analysis area was ~0.4 mm and the pass energy for the electron analysis was 50 eV. The base pressure of the analysis chamber was less than ~1 × 10^−9^ mbar. To obtain the O composition, we have carried out the curve fitting of the HR O 1s line-scan XPS spectrum using a Gaussian-Lorentzian peak shape after performing a Shirley background correction^37^.

### Electron-backscatter diffraction (EBSD)

We performed EBSD measurements using a FEI Quanta 3D FEG SEM equipped with AMETEK EBSD system to determine the crystallographic orientation of the Cu foils before and after graphene growth. During the measurements, the probe current, the accelerating voltage, and the incident angle of the beam were maintained at 16 nA, 15 kV, and 70°, respectively. The EBSD data were collected with a 0.25 μm step size and were analyzed using the TSL OIM Analysis 6 software for digitized inversed pole figures.

### HR plan-view TEM

Using a direct transfer method^69^, we transferred the graphene layer onto Au Quantifoil TEM grids. HR-TEM images and the corresponding selective area electron diffraction (SAED) patterns were taken by an FEI Titan cube G2 60-300 equipped with an image-aberration corrector and a monochromator. It was operated at an acceleration voltage of 80 kV to decrease the beam damage to the graphene layer, especially for detecting the graphene GBs.

### DFT calculations

We have performed DFT calculations on the penetration energy of single oxygen atom through a graphene sheet and on the lateral diffusion of O and H atoms from unsaturated C atoms. In this work, all calculations were performed using Vienna Ab-initio Simulation Package (VASP) based on the spin-polarized density functional theory^57,58^. We used ultra-soft pseudo-potential, and local density approximation of Ceperley and Alder type^70,71^. The electrons of wavefunctions were extended up to 500 eV of cutoff energy in a plane-wave basis set. The calculations were relaxed until forces on ions became less than 0.01 eV Å^−1^. For sampling Brillouin zone, the Monkhorst-Pack k-point grid was 5 × 5 × 1^72^. For the penetration calculations, we employed a 6 × 6 structure consisting of 72 carbon atoms for a free-standing graphene and a 4 × 4 graphene sheet (32 carbon atoms) and 3 layers of Cu(111) slab (48 Cu atoms) for a graphene on a Cu substrate. For the diffusion simulations, we employed the same structures but one carbon atom is missing to model the MV. The minimum energy pathway for the penetration and diffusion was examined by the nudged elastic band method (NEB)^73^.

### Data availability

The data that support the findings of this study are available from the corresponding authors upon request.

## Electronic supplementary material

Supplementary Information
