# Supplementary material for: Oxidation behavior of graphene-coated copper at intrinsic graphene defects of different origins
Source: Nat Commun. 2017 Nov 16;8:1549. doi: 10.1038/s41467-017-01814-8 (PMC5691087; doi:10.1038/s41467-017-01814-8)
Supplement: Supplementary file 1 — Supplementary Information [file 41467_2017_1814_MOESM1_ESM.pdf]

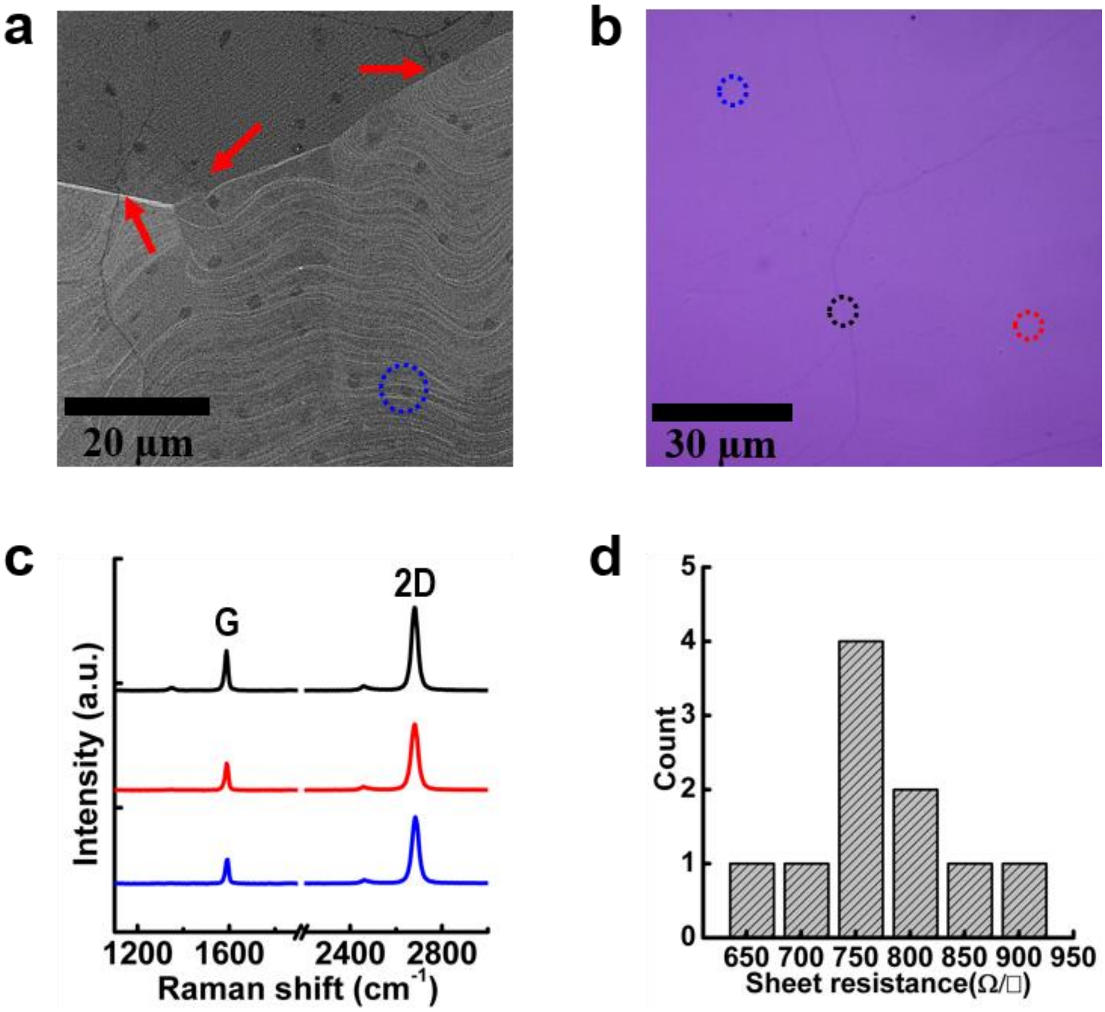

Supplementary Figure 1: Structural and electrical characteristics of the as-synthesized graphene. (a) A typical SEM image of graphene grown on a Cu foil at 1,000℃ for 30 min. Red arrows show several wrinkles that crossed the Cu grain boundary, and the blue-dotted circle indicates the region of a multilayer graphene flake. **(b)** An OM image of a graphene layer transferred onto SiO_2/Si using the conventional wet-etching process. **(c)** Raman spectra taken from blue, red, and black-dotted circles in (b). **(d)** Histogram of the sheet resistance of the transferred graphene layers on SiO_2/Si, as measured by the van der Pauw method.

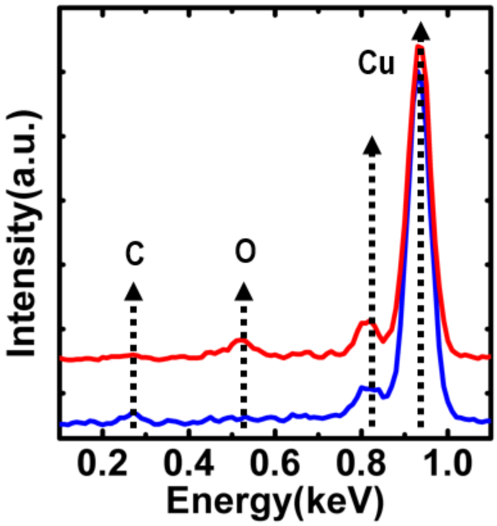

Supplementary Figure 2: Typical SEM-EDS spectra of the annealed Gr/Cu samples. The corresponding spectra were obtained from the red- and blue-dotted circles in Fig. 1b.

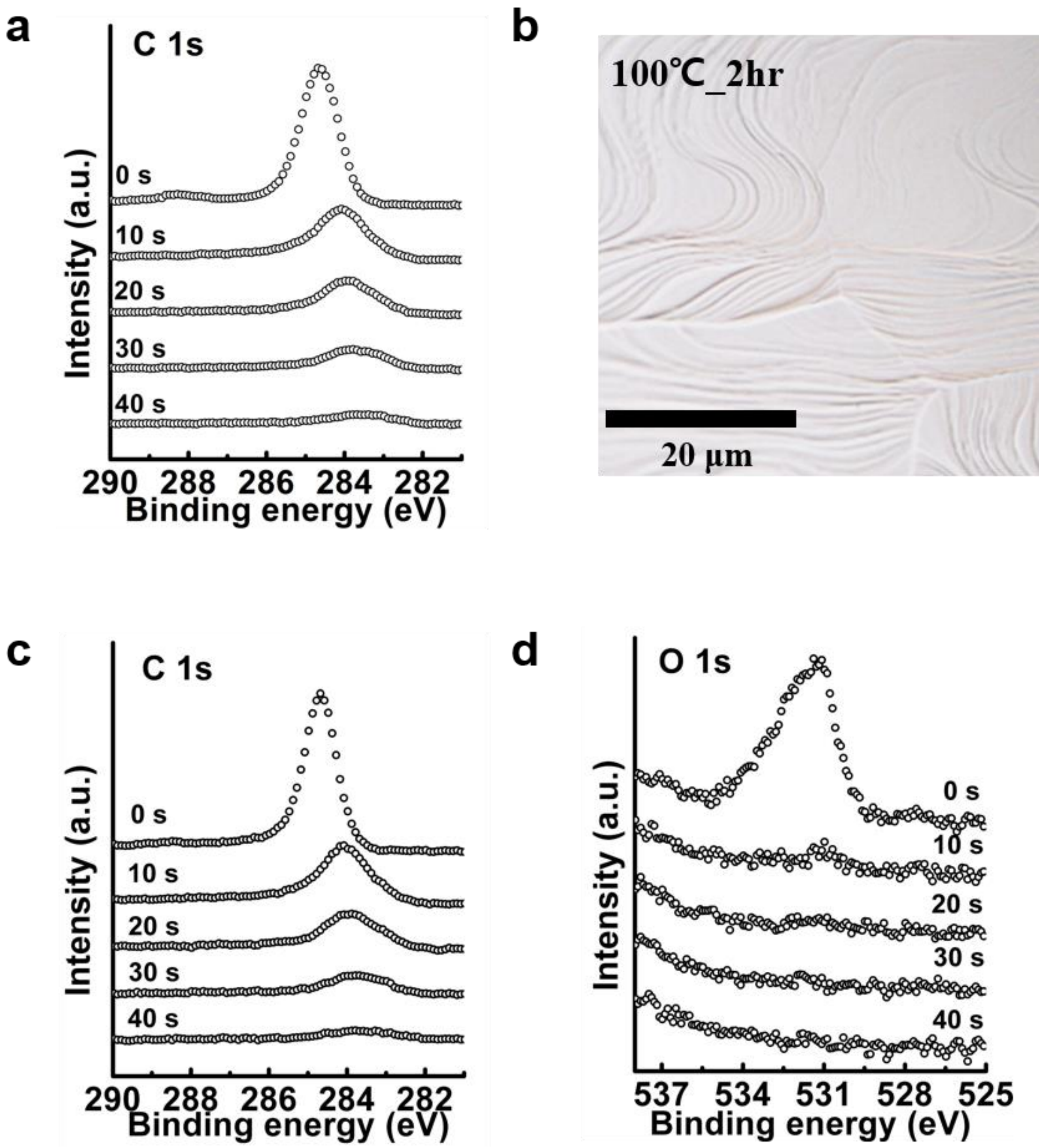

Supplementary Figure 3: Depth-profiling XPS spectra of the annealed Gr/Cu samples depending on the oxidation conditions. (a) High-resolution XPS C 1s line scans of the Gr/Cu sample after heating at 200℃ for 120 min in air, as a function of the sputtering time from 0 to 40 sec. **(b-d)** A typical OM image (b) of the Gr/Cu sample after heating at 100℃ for 120 min in air and the corresponding XPS (c) C 1s and (d) O 1s line scans, as a function of the sputtering time from 0 to 40 sec.

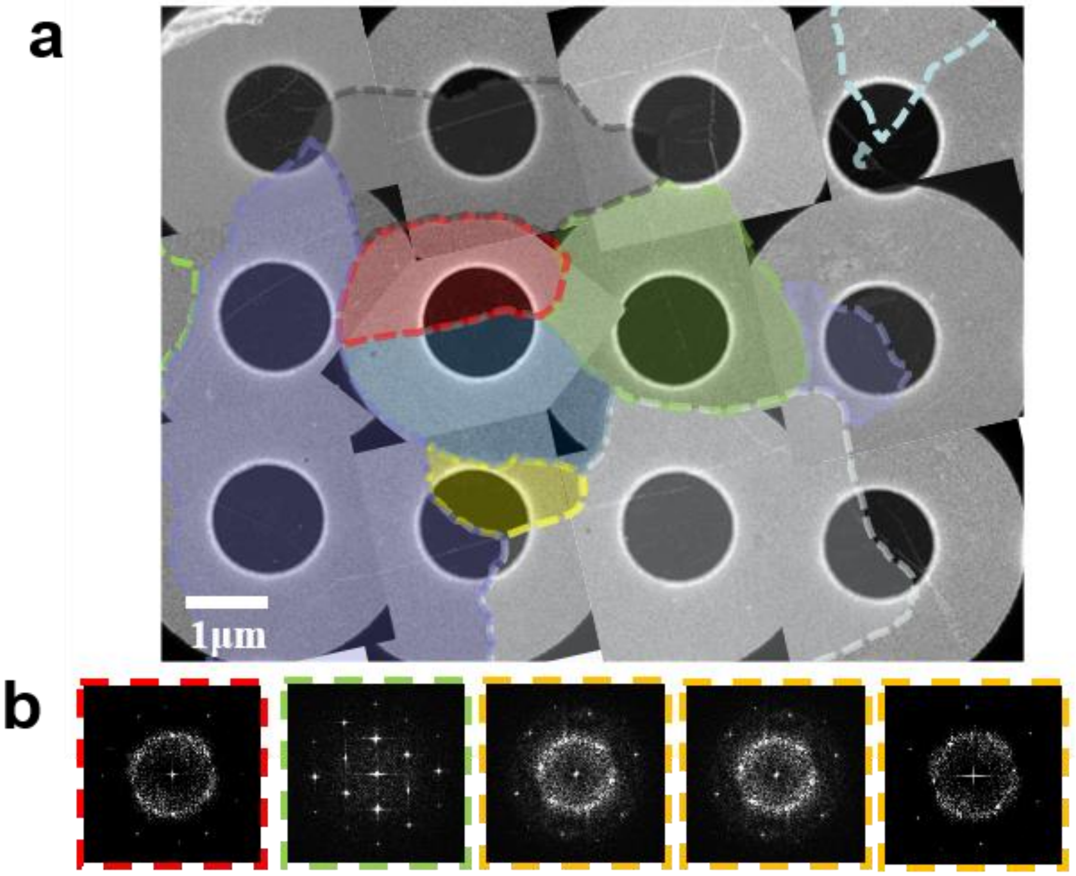

Supplementary Figure 4: Plan-view dark-field TEM image of a graphene sheet. (a) A representative plan-view TEM image of a graphene sheet grown on a Cu foil at 1,000℃ for 30 min and then transferred onto a TEM grid. The colour scheme highlights the different crystalline structures of the graphene grains. **(b)** FFT patterns of the coloured regions in (a).

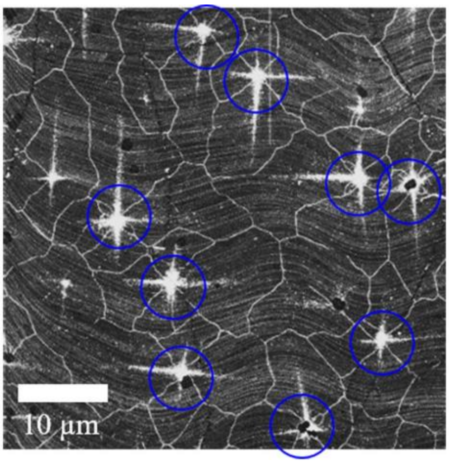

Supplementary Figure 5: Oxidation behaviour of graphene at the intra-granular GBs on the Cu(100) grains. A representative SEM image of the Gr/Cu sample annealed at 200℃ for 240 min in air.

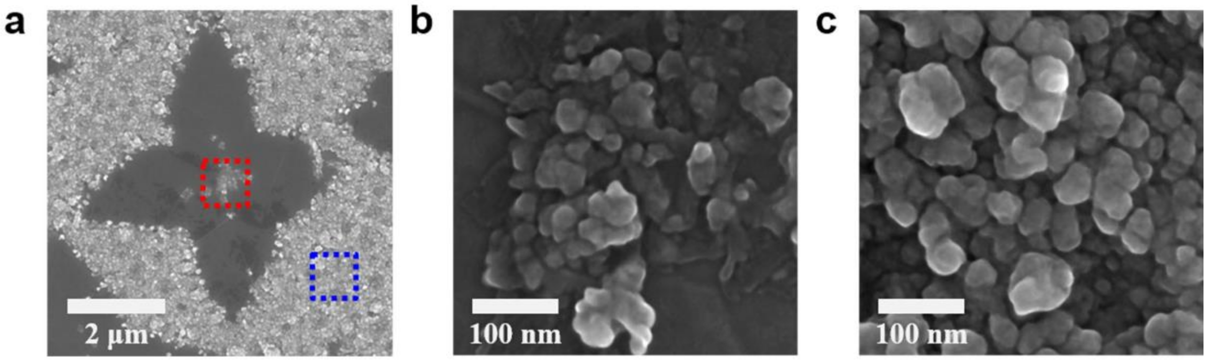

Supplementary Figure 6: Surface morphology of Gr/Cu samples after air oxidation process. (a) A typical low-magnification SEM image of Gr island grown on a Cu foil at 1,000°C for 1 min after annealing at 200°C for 70 min in air. High-magnification SEM images of **(b)** red- and **(c)** blue-dotted squares in (a), respectively.

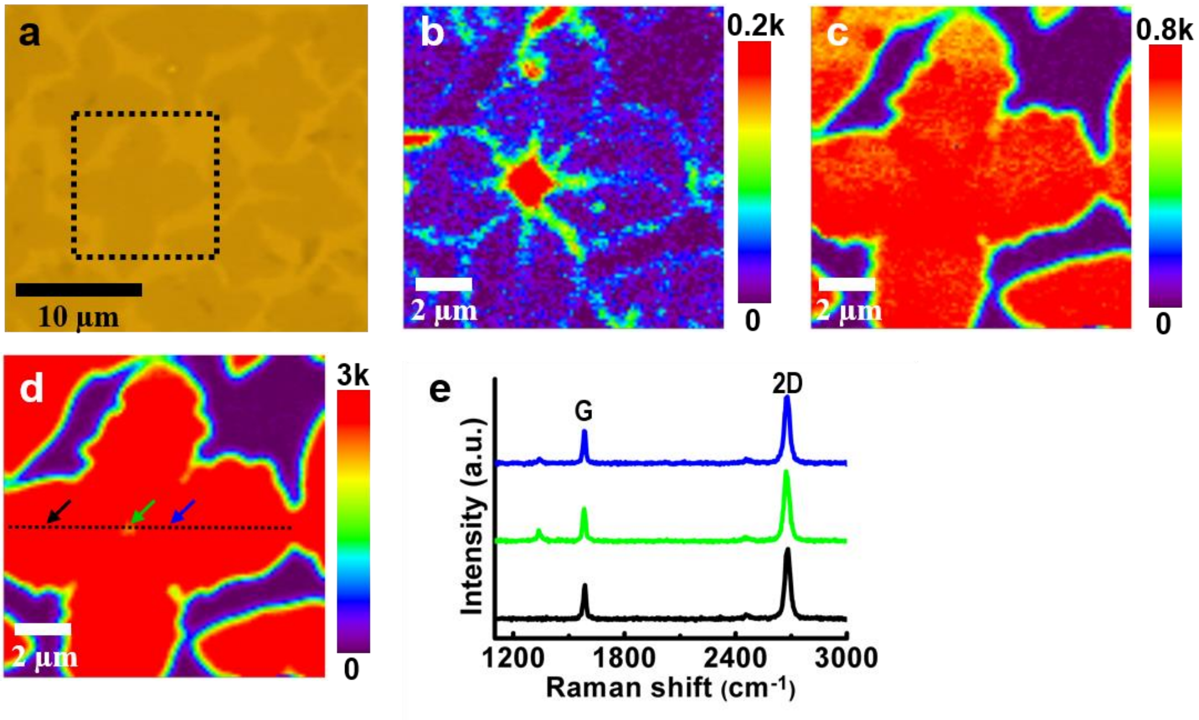

Supplementary Figure 7: Structural characteristics of the as-synthesized graphene islands without any oxidation process. (a) An OM image of the graphene islands transferred onto SiO_2/Si. **(b-d)** The corresponding Raman map images of the (b) D, (c) G, and (d) 2D bands. **(e)** Individual Raman spectra taken from coloured arrows along the black-dotted line in (d).

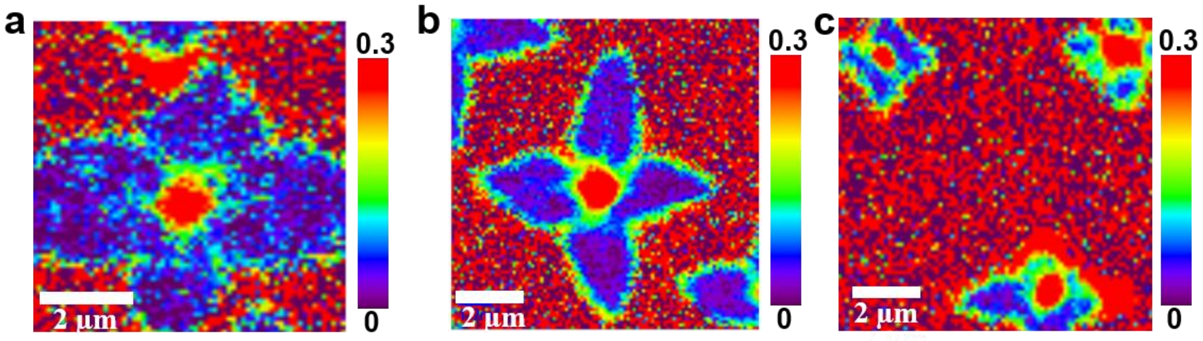

Supplementary Figure 8: Structural characteristics of the as-synthesized graphene islands with different sizes. Raman maps of the D/G band of graphene islands with the four-lobed shape that were grown on Cu foils at 1,000℃ for **(a)** 30 sec, **(b)** 15 sec, and **(c)** 5 sec and then transferred onto SiO_2(300 nm)/Si.

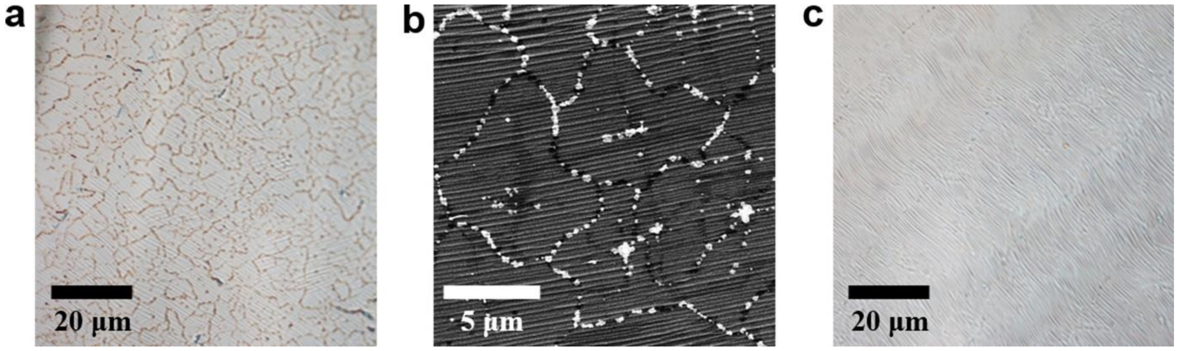

Supplementary Figure 9. Oxidation behaviour of Gr/Cu samples in a N_2 atmosphere. (a,b) Representative (a) OM and (b) SEM images of the Gr/Cu surface annealed at ~200 ℃ for 60 min in a wet-N_2 atmosphere (RH ≈50%). **(c)** A typical OM image of the Gr/Cu surface annealed at ~200 ℃ for 60 min in a pure N_2 atmosphere.

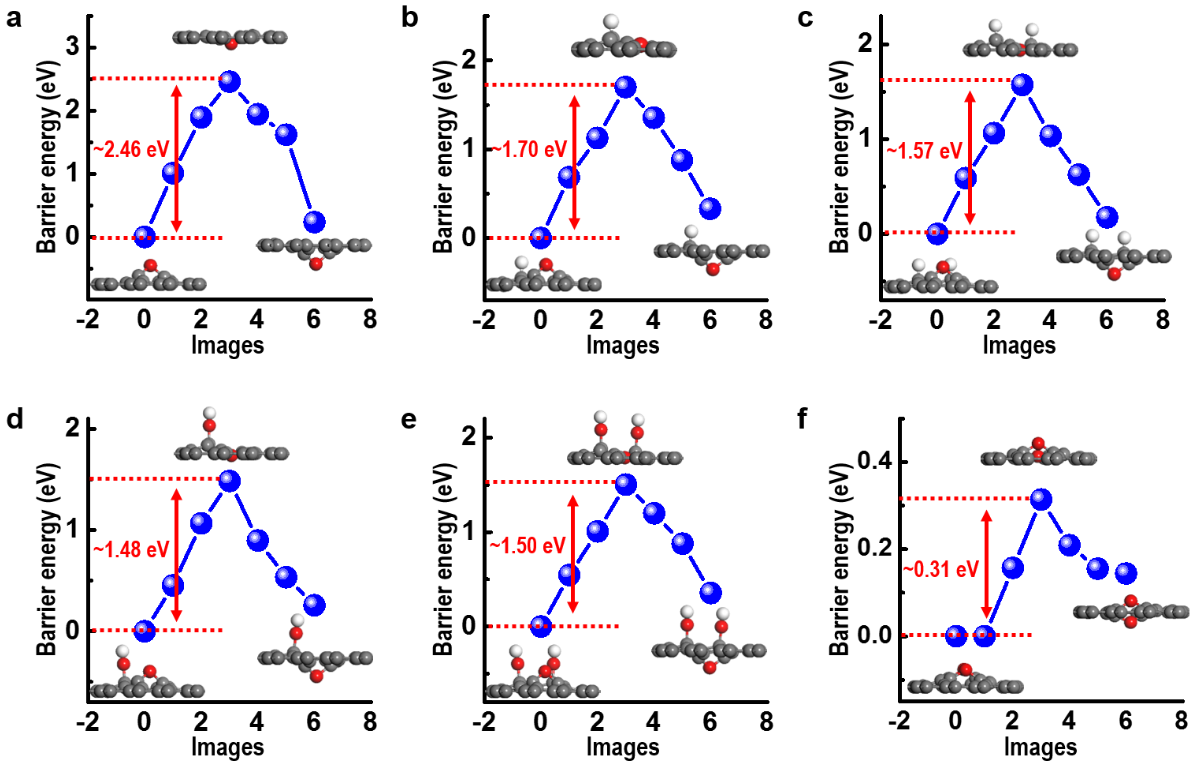

Supplementary Figure 10. Spin-polarized DFT calculation results for the inversion barrier of the O atom at the SW defect with different types of attached functional groups. (a) without (an epoxide) and **(b-f)** with (b) 1 H, (c) 2 H, (d) 1 OH, (e) 2 OH, or (f) 1 O.

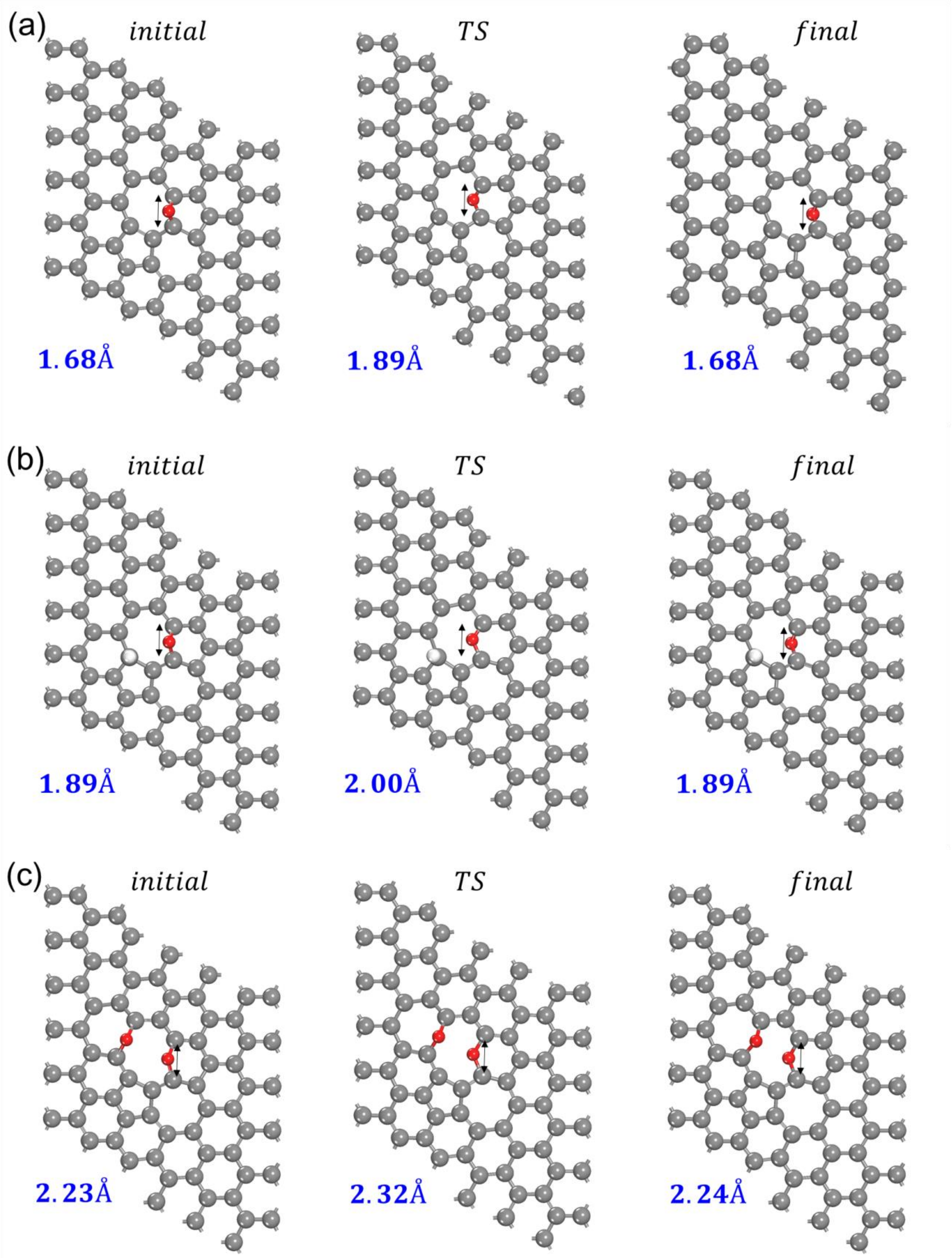

Supplementary Figure 11. **The initial, transition state, and final configurations of a single layer of graphene during the penetration of an oxygen atom (red) through the heptagon carbon ring of the SW defect: (a)** without any additional atom **(b)** with a single hydrogen atom (white), and **(c)** with another oxygen atom. The numbers below each configuration is the distance between two carbon atoms indicated by black arrows. It is clearly shown that an additional oxygen atom in (c) enlarges the distance between carbon atoms and thus makes larger space for the penetration. As a result, the energy barrier for the penetration is the smallest.

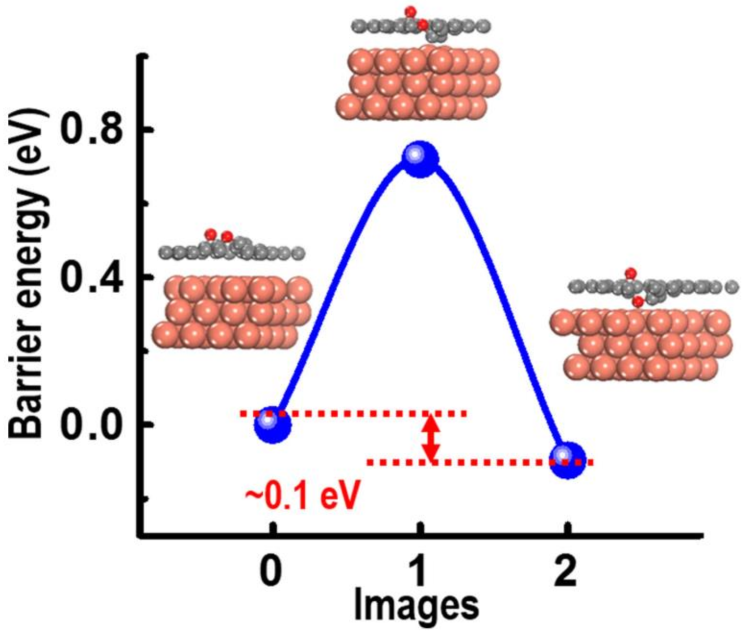

Supplementary Figure 12. Diagram of calculated transitional state energy of the O atom at a SW defect with a slab of Cu substrate when an additional O atom (red) is attached to sites around the epoxide.

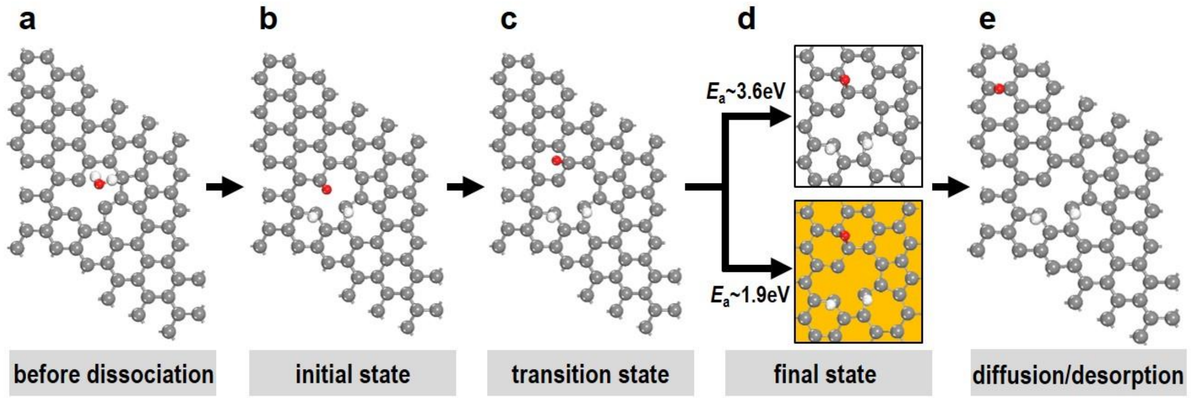

Supplementary Figure 13: Configurations of the O atom on a graphene mono-vacancy during the transformation. The white and red spheres indicate the H and O atoms, respectively. **(a, b)** A water molecule adsorbed on a mono-vacancy is dissociated into two H atoms and one O atom, resulting in the carbonyl group. **(c-e)** After the transition state, an epoxide is finally formed on the neighboring C ring, leaving the mono-vacancy intact. The presence of the underlying Cu greatly reduces the activation barrier of the dissociated O atoms into the neighboring C ring, as shown in (d).

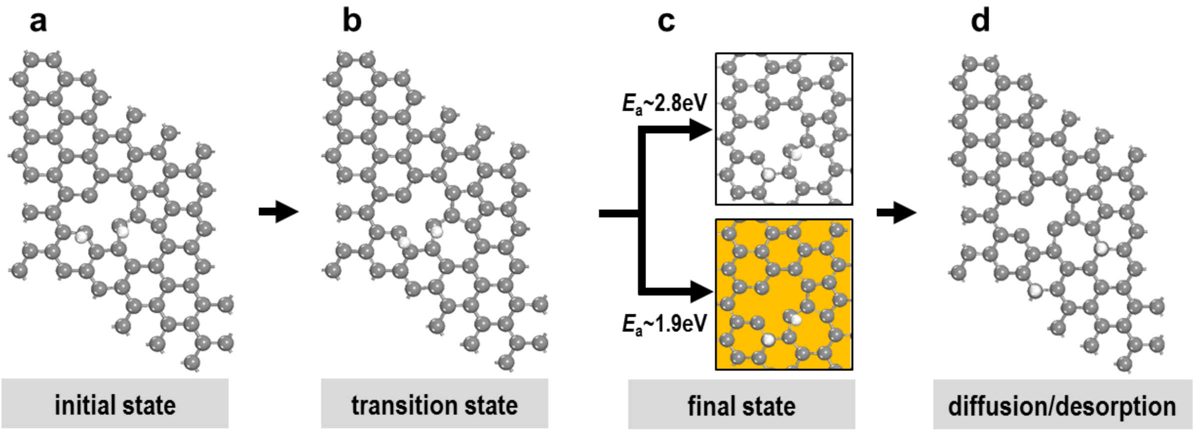

Supplementary Figure 14: Configurations of H atoms on a graphene mono-vacancy during the transformation. Configurations of the **(a)** initial, **(b)** transition, and **(c)** final state of H atoms (white spheres) on a graphene mono-vacancy during the transformation. The presence of the underlying Cu greatly reduces the activation barrier of the dissociated H atoms into the neighboring C ring, as shown in (c). **(d)** After the transition state, the graphene mono-vacancy returns to its original chemical state and thus it plays a role as the catalyst again.

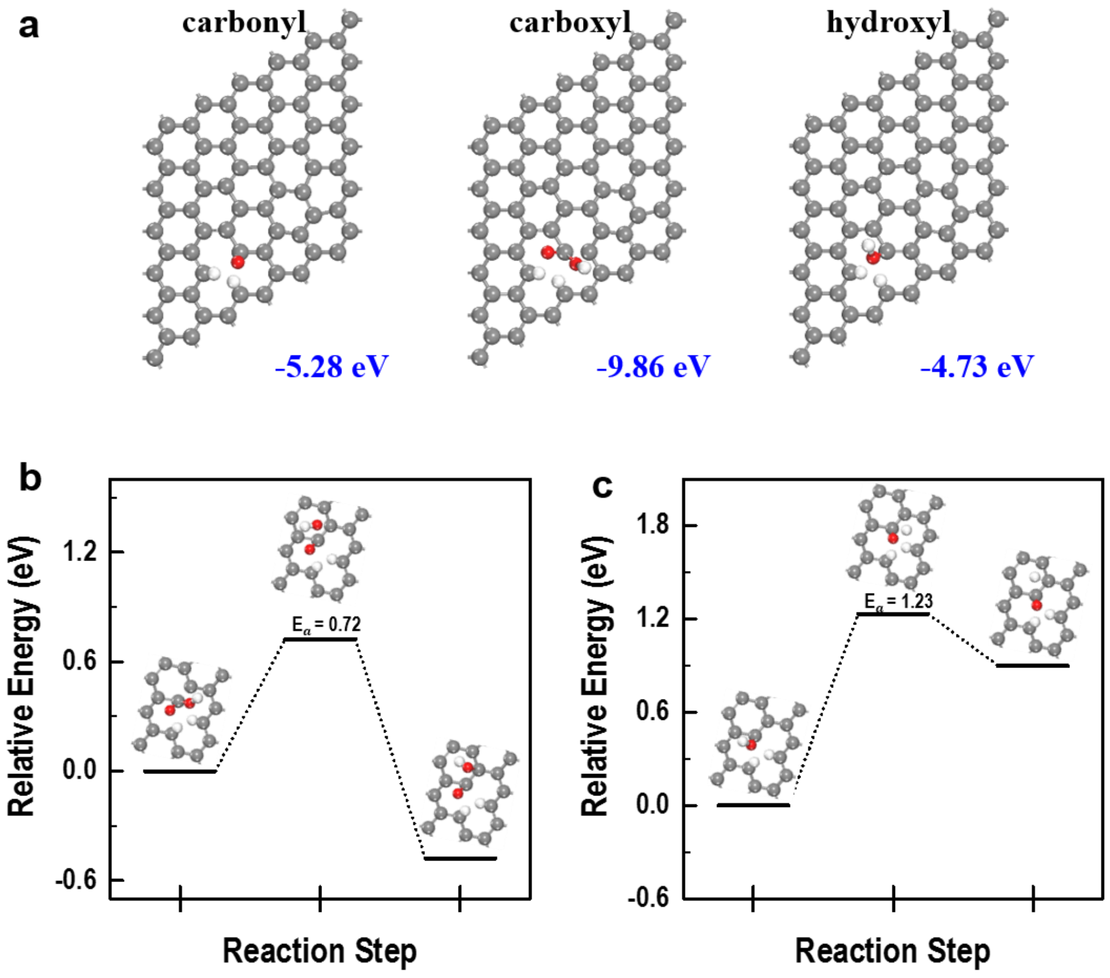

Supplementary Figure 15: Possible oxygen functional groups joining the oxidation mechanism after the dissociation process of a water molecule at graphene MV sites. (a) The stable (minimised) configurations of three functional groups at graphene MV sites: the carbonyl (left), carboxyl (middle), and hydroxyl (right) groups with their binding energies. Further possible reactions from the stable configurations to others are shown for the carboxyl group in **(b)** and the hydroxyl group in **(c)**. Both the carboxyl and hydroxyl structures are stable at their initial configurations, but the structures of the carboxyl and hydroxyl groups become the carbonyl configuration and an additional adsorbate (OH for carboxyl and H for hydroxyl) after reactions. The grey, white, and red spheres represent the C, H, and O atoms, respectively.

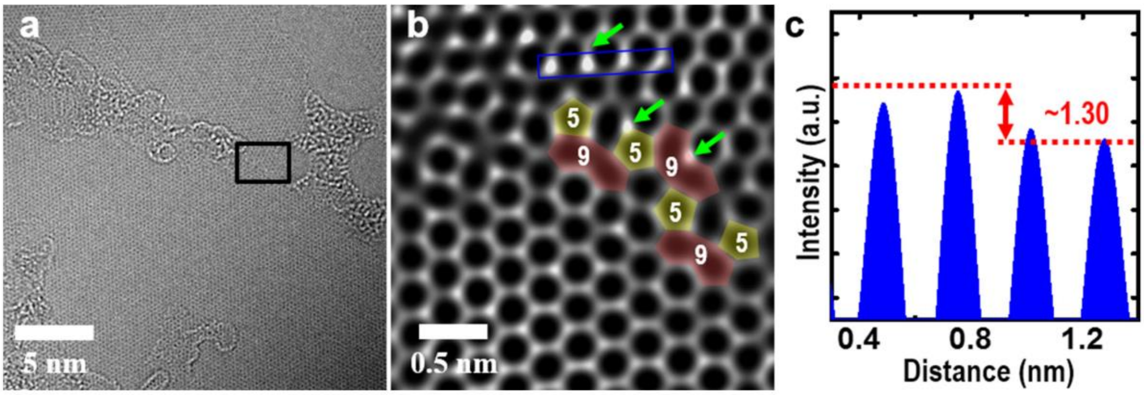

Supplementary Figure 16: Atomic structure of graphene GBs after the oxidation process. (a) Representative plan-view HR-TEM image of a Gr film that was annealed in air at 200℃ for 120 min and then transferred onto a TEM grid. **(b)** An atomic-resolution image of a GB of annealed Gr, indicated by a black square in (a). **(c)** Intensity line profile taken from a blue square in (b). The contrast ratio of brighter (indicated by green arrows in (b)) to surrounding carbon atoms is ~1.30, suggesting the substitution of oxygen atoms in graphitic lattice or the chemical bonding of oxygen atoms to the edge of carbon rings.

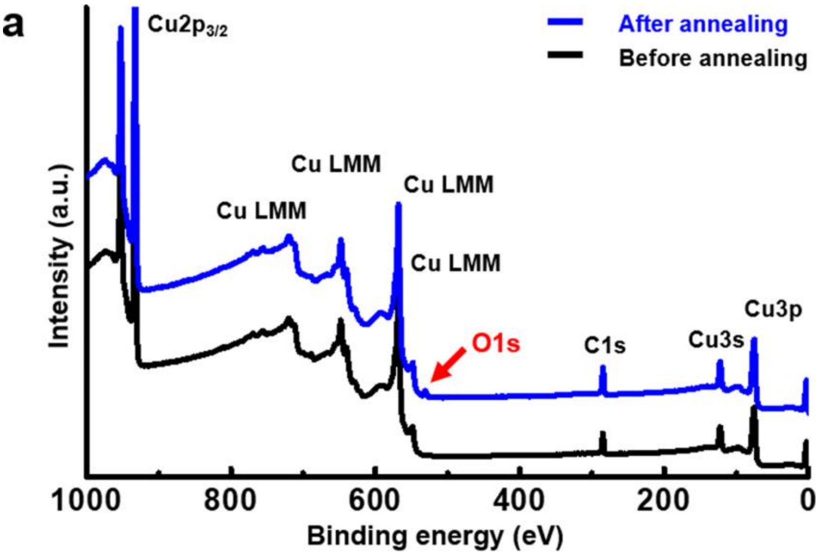

Composition (atomic percentage)

	Before annealing (%)	After annealing (%)
Cu	59.40	51.31
C	40.60	44.43
O	0	4.26
O/C		9.6×10^{-2} (8.8×10^{-2})

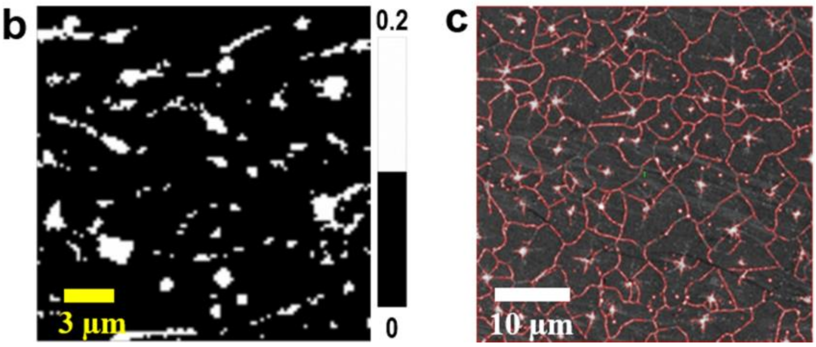

Supplementary Figure 17: Correlation between the O/C ratio in the annealed Gr/Cu samples and the estimated fraction of intrinsic graphene defects. (a) Representative XPS survey spectra of a Gr/Cu sample before (black line) and after (blue line) annealing at ~200° C for 120 min in air. The table shows the atomic composition of the samples obtained from each XPS survey spectrum. **(b)** Typical D/G Raman map image of graphene. The white and black regions in the D/G Raman map image correspond to the regions with magnitude of $I_D/I_G \geq 0.1$ and $I_D/I_G < 0.1$, respectively. **(c)** The SEM image of the annealed Gr/Cu sample taken from Fig. 1e in the manuscript. The fraction of Cu oxides (coloured by red lines) were measured using the image process software.

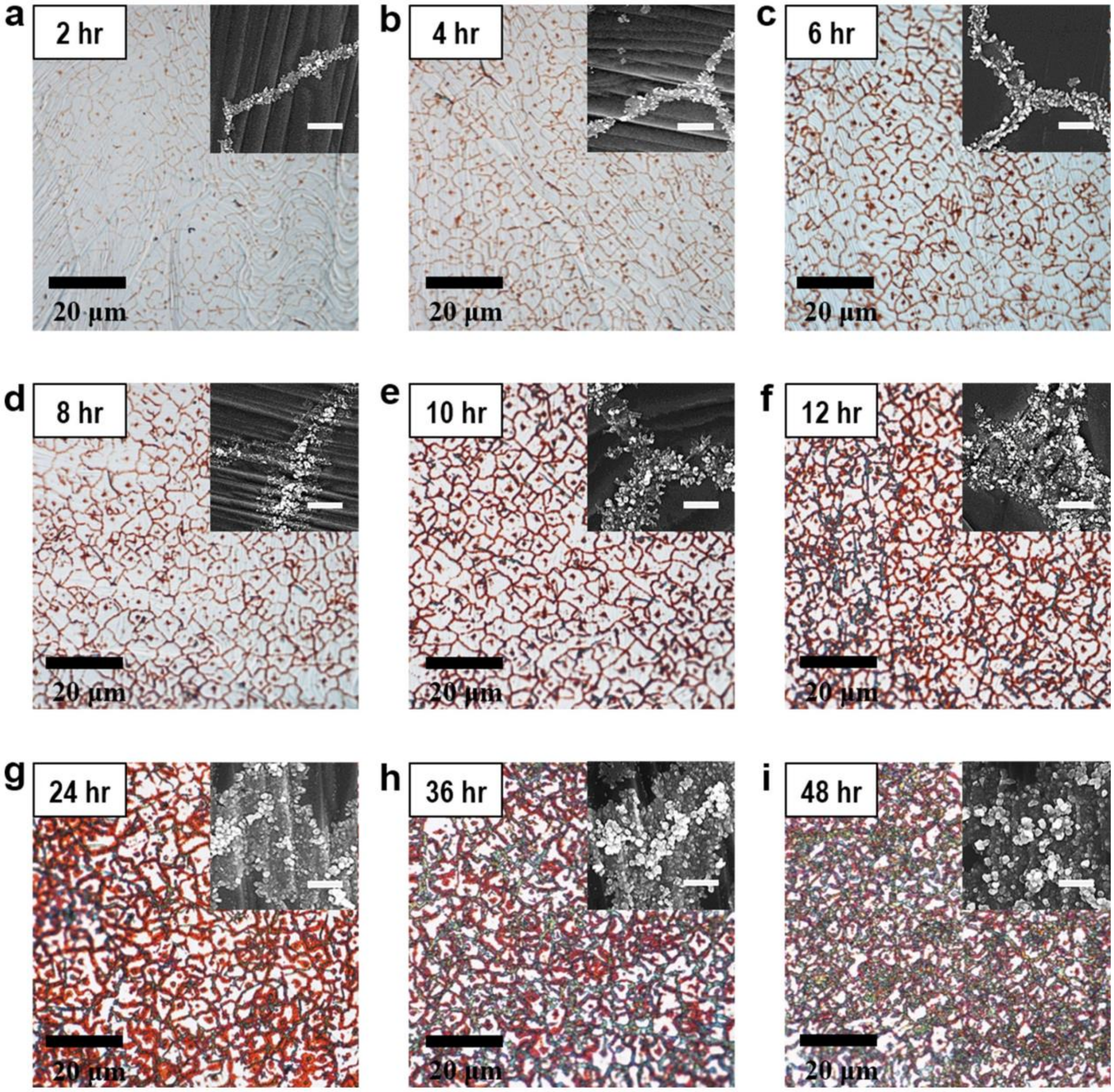

Supplementary Figure 18: Change in the oxidation line features of Gr/Cu samples depending on the annealing time. Typical OM images of the Gr/Cu samples annealed at 200℃ in air for **(a)** 2 h, **(b)** 4 h, **(c)** 6 h, **(d)** 8 h, **(e)** 10 h, **(f)** 12 h, **(g)** 24 h, **(h)** 36 h, and **(i)** 48 h. Insets in (a-i) are the corresponding SEM images of the oxidation line features originated from the inter-granular GBs. Scale bars are 500 nm.

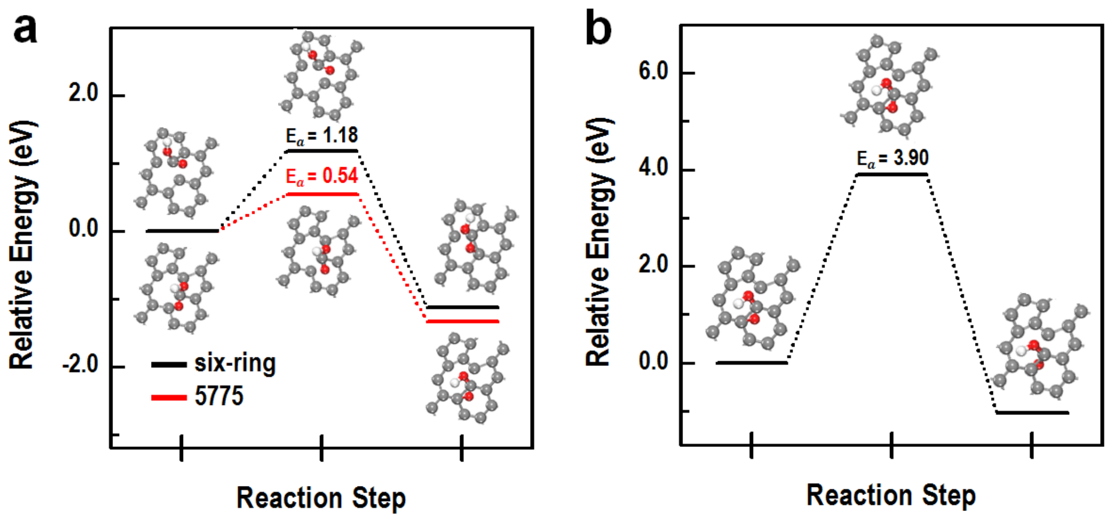

Supplementary Figure 19: Stability of carboxyl groups at the unzipped six-ring and SW defect structures of graphene. (a) The transformation energies from carboxyl (O=C-OH) to O-C-OH configurations on an unzipped six-ring defect (black line) and an unzipped SW defect (red line) of graphene. **(b)** The inversion energy barrier of an O atom from the O-C-OH configuration through an unzipped SW defect of graphene. The grey, white, and red spheres represent C, H, and O atoms, respectively. All models in the calculations include three layers of Cu slab coated by graphene structures.

Supplementary Note 1: Possible oxygen functional groups joining the oxidation mechanism after the dissociation process of a water molecule at graphene MV sites. As described in the main text, we examined the possibility that other functional groups join the oxidation mechanism, especially after the dissociation process of a water molecule at graphene MVs. For this, we considered carboxyl (O=C-OH) and hydroxyl (C-OH) groups, as shown in Supplementary Fig. 15b-c, which were detected by XPS in the experiment (Figs. 1c and 4f in the main text). The carboxyl group can be generated by the combination of a carbonyl group at graphene MV (marked by the red dotted lines in Fig. 5a) and dissolved H and O atoms from the water molecule. The C atom of the carbonyl group that was originally participating in the O=C bond breaks the bond with the nearby C atom and interacts chemically with the H and O atoms, resulting in a carboxyl group. The initial configuration of the optimised carboxyl group at graphene MV is as shown in Supplementary Fig. 15b. This carboxyl group needs to be transformed to some other configurations which can release a single O atom to join in further reactions for the oxidation process. We found through DFT and NEB calculations that the carboxyl group could be decomposed to a carbonyl group and an OH molecule with an energy barrier of ~0.72 eV, which is the lowest value we were able to obtain, as shown in Supplementary Fig. 15b. (Other considered pathways are not shown here either because they were unstable or they exhibited a higher energy barrier). The dissolved OH molecule from the carboxyl group is positioned and stabilised at top of the C atom, as shown in the final configuration in Supplementary Fig. 15b, and it can diffuse outward from the MV site with an energy barrier of ~0.5 eV [Gűrel *et. al., J. Phys.*: *Condense. Matter* **5**, 435304 (2013)]. Once the outward diffusion of the OH molecule has occurred, the final state of the carboxyl group returns to the same configuration in which a carbonyl group and two H atoms exist, which results from the direct dissociation of a water molecule at MV; the configuration is as shown in the red dashed line in Fig. 5a. This means that forming a carboxyl group at a graphene MV site is energetically stable, but it can transform to a carbonyl group and thus further contribute to oxidation processes.

Next, as a stable intermediate at graphene MV, the hydroxyl group can also be formed by the chemical bond between the carbonyl group and a dissolved H atom. Again, although the hydroxyl group can contribute to the oxidation process by releasing O atoms, the hydroxyl group needs to be transformed to some proper configuration. As seen in Supplementary Fig. 15c, the H atom can diffuse from the top of the O atom to top of a neighbouring C atom with a relatively high diffusion barrier of ~1.23 eV. Once the dissolved H atom diffuses, the final

state becomes the same carbonyl configuration as shown by the red dashed line in Fig. 5a. Similar to the carboxyl group, the stable hydroxyl group at graphene MV can only contribute to the oxidation process after it has transformed to a carbonyl group and a single H atom.

The discussion above allows us to conclude that the energetically stable configurations of the carboxyl and hydroxyl groups can be formed at graphene MV. However, they should transform to proper configurations which O atoms can be generated from. Relying on our DFT and NEB calculations, we found that it seems hardly possible that the carboxyl and/or hydroxyl groups can release O atoms unless they have first transformed into the carbonyl group and other molecules. Once they have transformed to the carbonyl group, the O atom in the carbonyl group can contribute to the oxidation process via the same procedure (Step 2-5) as displayed in Fig. 5a. Therefore, we believe that the carbonyl groups play a key role in the successive production of O atoms by the dissociation of water molecules at graphene MV.

Supplementary Note 2: Correlation between the O/C ratio in the annealed Gr/Cu samples and the estimated fraction of intrinsic graphene defects. As discussed in the main text, we have compared the O/C ratio of the annealed Gr/Cu samples with the fraction of intrinsic defects in graphene. First, we measured the O/C ratio of graphene after air oxidation at ~200 ^{o}C for 120 min from XPS survey spectra (Supplementary Fig. 17a). The O/C ratio of the annealed graphene was ~9.6×10^{-2}, which was decreased to ~8.8×10^{-2} by excluding the O content corresponding to the CuO/Cu_2O phase. Then, we evaluated the fraction of intrinsic graphene defects using the D/G Raman map image (Supplementary Fig. 17b). The fraction of intrinsic defects in graphene was defined as A_{defect} /A_{G} (A_{defect}: area of the regions with magnitude of $I_{\mathrm{D}}/I_{\mathrm{G}} \geq 0.1$ in the D/G Raman map image, A_{G}: total area of the D/G Raman map image) and the measured value of the A_{defect} /A_{G} was ~7.9×10^{-2}, which was similar to the O/C ratio (~8.8×10^{-2}) of the annealed graphene obtained from the XPS studies. We suggest that these analogous values may come from surface contamination of the air-annealed Gr/Cu samples by adsorbing hydrocarbon and/or carbon oxide molecules [Li *et. al.*, *Nat. Mater.* **12**, 925-931 (2013)], which contributes to the increased O/C ratio in the XPS result, although Raman spectroscopy generally overestimates the microscopic defect density in a graphene sheet because of a significantly large laser spot size (~1 μm) beside various microscopic graphene defects. To further investigate it, we analyzed the fraction of oxidized Cu regions on the Gr/Cu surface after the annealing process using the SEM image. As shown in Supplementary Fig. 17c, the fraction of Cu oxides on the Gr/Cu surface (~9.0×10^{-2}) was also analogous to the O/C ratio of the annealed graphene in air. These results imply that, after the annealing process, defective regions in the graphene are selectively functionalized by oxygen with various configurations, such as carbonyl and carboxyl groups.

Supplementary Note 3: Stability of carboxyl groups at the unzipped six-ring and SW defect structures of graphene. As discussed in the main text, abundant carboxyl groups were detected by XPS in the experiment, and we have proved that carboxyl groups can be formed as energetically stable products at graphene MVs in our DFT calculations. In addition, we have provided much clear evidence and explanations about how the dissociation of water molecules takes place at atomic defects on graphene sheets such as MVs and the inversion of oxygen atoms occurs at GBs of graphene via both experiments and computation. Then, there are a few minor but necessary questions: "why do carboxyl groups exist as abundantly as was detected in the experiment?" and "is there any possibility that these carboxyl groups contribute to the oxidation process?" In fact, these two questions are closely related to each other in the manner of how significantly the abundant carboxyl groups contribute to the entire oxidation mechanism. Note that a partial explanation of the first question is that carboxyl groups are stable at MVs and they are not directly involved in the water dissociation. We have answered both questions by calculating the stability of carboxyl groups at defects other than MVs (Supplementary Fig. 19a) and the possibility of oxygen inversion from carboxyl group through atomic defects in graphene (Supplementary Fig. 19b).

We have examined the stability of carboxyl groups by considering the disconnection between C atoms' so-called unzipping instead of vacancies for two cases: perfect six-ring and SW defect structures. In both cases, two C-C bonds of graphene are broken so that an unzipped six-ring defect (from the perfect six-ring structure) and an unzipped SW defect (from the SW defect structure) are generated. Then, carboxyl groups are formed at one of the broken C atom sites by making a O=C-OH bond structure. We display their optimised structures as the initial configurations in Supplementary Fig. 19a. The binding energies of carboxyl groups are found to be ~3.99 and ~5.76 eV for the unzipped six-ring and SW defects, respectively. Therefore, energetically stable carboxyl groups can be formed even at the six-ring and SW defects of graphene. These carboxyl groups can be transformed to the more stable O-C-OH configurations, which is described as the final state in Supplementary Fig. 19. The calculated transformation energies are ~1.18 eV and ~0.54 eV for the unzipped six-ring and SW defects, respectively.

In addition, we focused on the possibility of inverting the O atom at O-C-OH onto unzipped SW graphene, as shown in Supplementary Fig. 19b, because the unzipped 5775 graphene with the carboxyl group was more stable than the six-ring configuration. As

summarised in Supplementary Fig. 19b, the estimated inversion energy of the O atom from O-C-OH state was very high at ~3.9 eV. It is noticeable that the O-C-OH structure made it difficult to contribute to the oxidation of the Cu substrate.
